# Extensive dynamic changes in the human transcriptome and its circadian organization during prolonged bed rest

**DOI:** 10.1016/j.isci.2024.109331

**Published:** 2024-02-22

**Authors:** Simon N. Archer, Carla Möller-Levet, María-Ángeles Bonmatí-Carrión, Emma E. Laing, Derk-Jan Dijk

**Affiliations:** 1 Surrey Sleep Research Centre, Faculty of Health & Medical Sciences, University of Surrey, Guildford, UK; 2 Bioinformatics Core Facility, Faculty of Health & Medical Sciences, University of Surrey, Guildford, UK; 3 Chronobiology Laboratory, Department of Physiology, University of Murcia, Murcia, Spain; 4 Ciber Fragilidad y Envejecimiento Saludable, Instituto de Salud Carlos III, 28029 Madrid, Spain; 5 Department of Microbiology, Faculty of Health & Medical Sciences, University of Surrey, Guildford, UK; 6 UK Dementia Research Institute Care Research & Technology Centre, Imperial College London & University of Surrey, Guildford, UK

**Keywords:** Medical research, Human specimen, Expression study, Transcriptomics

## Abstract

Physiological and molecular processes including the transcriptome change across the 24-h day, driven by molecular circadian clocks and behavioral and systemic factors. It is not known how the temporal organization of the human transcriptome responds to a long-lasting challenge. This may, however, provide insights into adaptation, disease, and recovery.

We investigated the human 24-h time series transcriptome in 20 individuals during a 90-day constant bed rest protocol. We show that the protocol affected 91% of the transcriptome with 76% of the transcriptome still affected after 10 days of recovery. Dimensionality-reduction approaches revealed that many affected transcripts were associated with mRNA translation and immune function. The number, amplitude, and phase of rhythmic transcripts, including clock genes, varied significantly across the challenge.

These findings of long-lasting changes in the temporal organization of the transcriptome have implications for understanding the mechanisms underlying health consequences of conditions such as microgravity and bed rest.

## Highlights

• Largest time series human transcriptomic dataset collected during 90 days bed rest
• Extensive and dynamic disruption to the transcriptome temporal organization
• 91% of transcripts affected, including major disruption to rhythmic transcripts
• Processes affected included muscle function, immune response, and translation

## Graphical abstract

**Figure:**
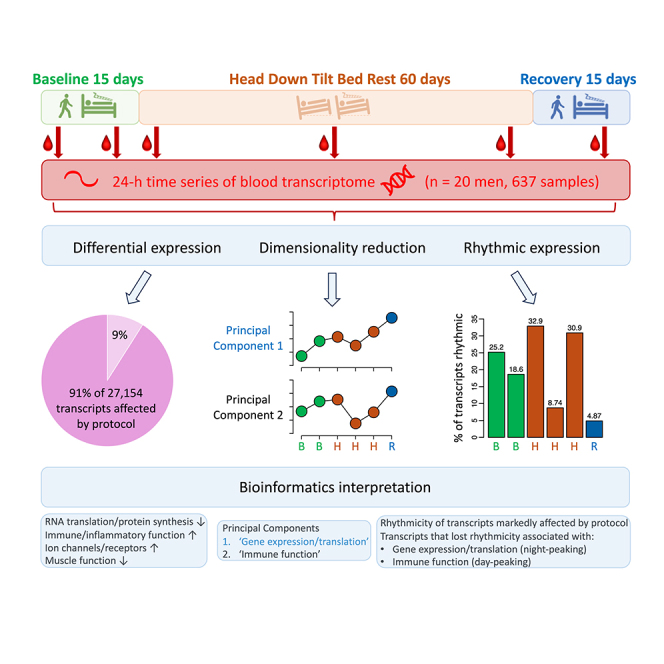

## Introduction

Twenty-four-hour rhythmicity in gene expression is ubiquitous: it is present in all eukaryotic and some prokaryotic species and in nearly all tissues, organs, and cells of these organisms.^1^ This rhythmicity in gene expression generates variation in molecular and physiological processes in central and peripheral tissues to optimize adaptation to the organism’s temporal niche, e.g., nocturnal or diurnal.^2^ This rhythmicity is driven by a core circadian molecular mechanism but also by environmental, systemic, and behavioral factors, which may affect either the core circadian machinery or the expression of genes outside the core circadian machinery.^3^ The light-dark and endocrine cycles are well-known examples of environmental and systemic factors, respectively. The “sleep-wake” cycle is a prominent behavioral factor which comprises several associated components, such as the light-dark and feeding-fasting cycles, but also alternation between the upright and supine posture.

Circadian and diurnal rhythmicity of gene expression in tissues and organs has also been shown to be altered in response to immune challenges,^4^ extension of the wake period,^5^ mistimed sleep,^6^ acute total sleep deprivation and repeated partial sleep deprivation,^7^ and behavioral manipulations such as timed feeding and fasting.^8^ These changes in expression rhythms are not limited to genes outside the core circadian machinery but include core clock genes, at least in tissues peripheral from the central circadian clock in the suprachiasmatic nuclei. The interpretation of changes in rhythmicity in response to challenges may be 2-fold. On the one hand they may represent an adaptive response of tissues and organs to the challenge, e.g., changing rhythmicity in metabolic and associated endocrine profiles in response to altered timing of feeding. On the other hand, they may represent pathways by which certain challenges are associated with negative outcomes such as poor health. For example, mistimed sleep, as occurs in shift work, is associated with poor health outcomes,^9^ and changes to the expression rhythms of the transcriptome in tissues and organs may underlie these outcomes.

Although the relevance of circadian rhythmicity for medicine is increasingly recognized,^10^^,^^11^ in most investigations of the response of the transcriptome to challenges the rhythmic organization of the transcriptome has not been taken into account. In those investigations in which rhythmicity was considered the challenges were in general short lasting.^12^^,^^13^^,^^14^ A description of how the 24-h organization of the transcriptome changes in the course of a longer challenge may provide new insights into the functional and adaptive significance of the temporal organization of the transcriptome, as well as mechanisms underlying adverse consequences of such challenges. Here, we assessed 24-h rhythmicity in the human blood transcriptome before, during, and after 60 days of constant bed rest head-down tilt (HDT). This Earth-based model simulates in healthy participants the effects of microgravity experienced by astronauts, which affect multiple physiological processes and mechanisms^15^ which during exposure to normal gravity maintain physiological homeostasis. For example, during exposure to normal gravity blood pressure and blood supply to the brain are kept within a normal range by activation of the autonomic nervous system when we change from the supine to the upright posture, but this cyclic challenge is absent during microgravity. Spaceflight has been shown to reduce sleep quality,^16^ and spaceflight-induced sleep deficiency^16^^,^^17^ was associated with less than 6 h sleep durations, which we have previously shown reduced both the amplitude and rhythmicity of the human blood transcriptome.^7^ Spaceflight also affected the amplitude and phase timing of circadian rhythms in astronauts,^16^^,^^18^^,^^19^ and we have shown that mistimed sleep and circadian rhythms had a large disruptive effect on the temporal organization of the human blood transcriptome.^6^ Physiological circadian rhythm disturbance was also demonstrated in a 7-day HDT bed rest protocol.^20^ The protocol is also relevant as a model for reduced use of the skeletal musculature and associated atrophy of musculoskeletal mass that occurs because of long-term confinement to bed and aging.

Whether this HDT bed rest protocol and associated changes in the rhythmicity of the autonomic nervous system and activity affect the temporal dynamics of the peripheral transcriptome, including transcripts of core circadian genes, has not been reported before. We took a bulk-transcriptome systems approach and analyzed levels of expression and rhythmicity of all transcripts in whole blood at baseline, during and after 60 days of bed rest HDT in 20 male volunteers. Whole-blood samples consist of many different cell types with a variety of functions, and this systems approach is more feasible and also may better capture the complexity of adaptive responses compared with a single cell type approach. We applied dimensionality reduction to the participant-centered transcriptome to identify groups of transcripts in which the rhythmicity and levels of expression demonstrate a correlated response to time of day and the prolonged challenge. We also analyzed the response of core circadian genes, which are expressed in all nucleated blood cell types. The results demonstrate marked non-monotonic changes in the number and amplitude of rhythmic transcripts including transcripts of core circadian genes, and differential expression of transcripts. These changes can be interpreted as a succession of adaptive responses that affect the timing and amplitude of processes such as translation and immune function, and up- and down-regulation of pathways associated with muscle proliferation and interferon/interleukin signaling.

## Results

### Effect of the protocol and time of day on transcript expression

**Figure 1:**
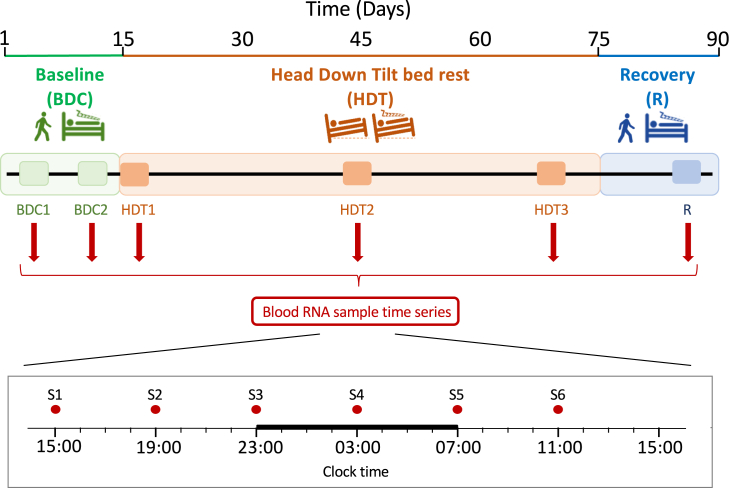
Study protocol 2 weeks of baseline (BDC, days 1–14) was followed by 60 days of head-down tilt constant bed rest (HDT, days 15–75, and 2 weeks of recovery (R, days 76–90). Time series 4-hourly blood samples were collected at 6 sample points at the clock times indicated (S1–S6) during 24 h sessions that occurred twice in baseline (BDC1, days 2/3; BDC2, days 10/11), three time during head-down tilt (HDT1, days 15/16; HDT2, days 41/42; HDT3, days 68/69), and once during recovery (R, days 86/87).

All 20 healthy young men that were recruited to the study completed the study protocol which consisted of a 2-week baseline period confined to the study clinic with a normal sleep/wake cycle, followed by 60 days of constant bed rest in a −6-degree HDT position, ending with 2 weeks of recovery in the same conditions as for the baseline period (Figure 1). A total of 701 blood samples were successfully collected during 6, 24-h sampling sessions (6 samples per 24 h) throughout the protocol, and, of those, 637 were used for the time series transcriptome analyses. Linear mixed model analysis revealed that 24,716 transcripts out of 27,154 (91%) changed significantly (Benjamini-Hochberg [BH]-corrected p < 0.01) across the protocol (main effect, 6 sampling sessions [baseline data collection BDC1, BDC2; HDT HDT1, HDT2, HDT3; recovery, R]); 18,973 (70%) transcripts changed across the 24 h day (although not necessarily in a rhythmic manner); and a significant interaction between time of day and sampling session was observed for 49 (0.18%) transcripts.

### Transcripts were differentially expressed throughout the protocol

**Figure 2:**
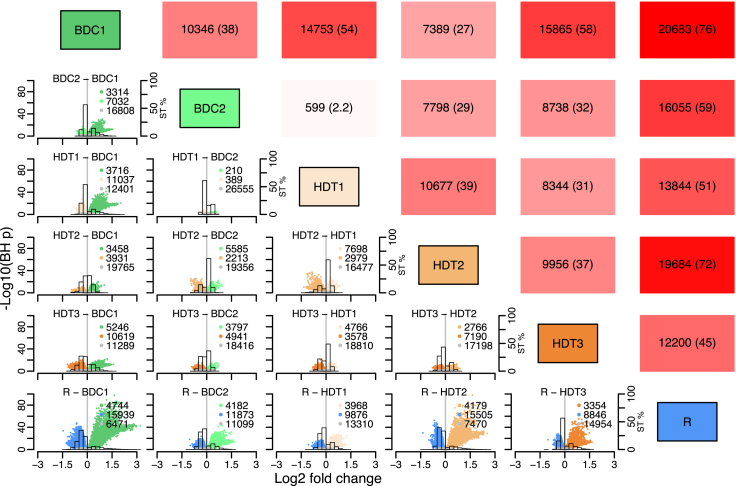
Effect of protocol on differential expression To assess the differential expression among protocol segments, we conducted a linear mixed model analysis, highlighting the significant fold change differences (BH-corrected p < 0.01) attributed to the main effect of sampling session. Diagonal boxes indicate all pairwise comparisons between sampling sessions. Upper triangle presents the total number of differentially expressed transcripts per pairwise sampling session comparison (% in brackets) with darker red indicating more transcripts. The total number of transcripts evaluated is 27,154. Lower triangle presents volcano plots (statistical significance vs. magnitude of change) for all pairwise sampling session comparisons. Sample color indicates the sampling session with the higher expression level in the pairwise comparison. Samples in gray are not significant. The legend in each plot shows the total number of transcripts per color. Histograms represent the percentage of significant transcripts (ST%, right vertical axis).

Within the many thousands of transcripts that changed during the protocol there were more down-regulated transcripts in session comparisons than up-regulated, some with very large fold changes (e.g., BDC1 vs. R fold changes of −3) (Figure 2; Data S1). Of notable exception, there were many more up-regulated transcripts in HDT2 compared with BDC2 and HDT1. Up- and down-regulated transcripts were observed not only during the bed rest HDT period but also from the beginning to the end of the baseline, i.e., the period during which participants were confined to the research facility but did get out of bed during the wake period. 38% of the transcriptome was differentially expressed when comparing BDC1 with BDC2 (Figure 2). Surprisingly, there were very few acute changes in gene expression when exposed to the challenge (HDT1 compared with BDC2), but there was a large change in gene expression (39%) with prolonged exposure to bed rest (HDT1 vs. HDT2). The largest number of differentially expressed transcripts (76%) was observed when comparing BDC1 with R, and this indicates that gene expression at 10 days of recovery did not return to resemble baseline levels. Indeed, 15,939 transcripts were down-regulated and 4,744 up-regulated in R compared with BDC1, and 11,873 and 4,182 were down- and up-regulated, respectively, compared to BDC2.

### Gene set enrichment analysis (GSEA) identified gene ontology (GO) terms associated with differential expression

**Figure 3:**
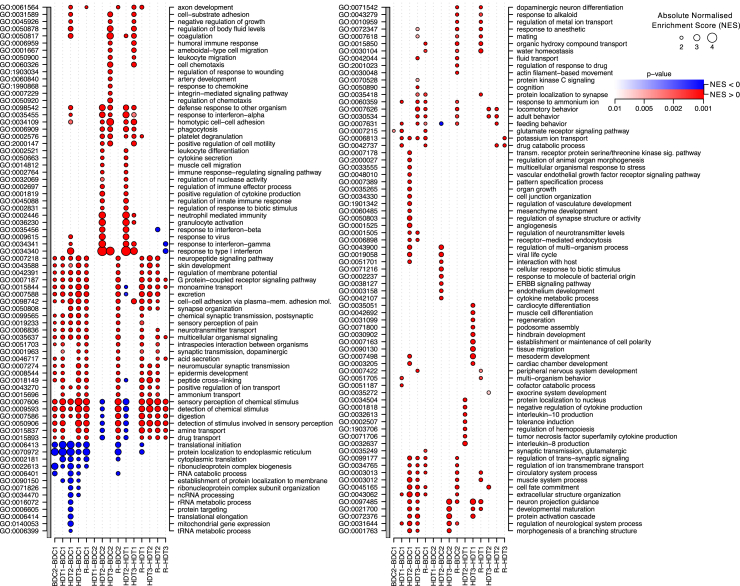
Functional enrichment of differentially expressed genes Gene Ontology (GO) biological process enrichment of differentially expressed genes based on gene set enrichment analysis (GSEA). Vertical axes display GO terms (left) and their description (right). Horizontal axis indicates comparison between sampling sessions (e.g., red circle in BDC2-BDC1 indicates enrichment of the term in up-regulated genes in BDC2 compared with BDC1). Circle diameter corresponds to the normalized enrichment score (NES, enrichment score normalized to mean enrichment of random samples of the same size); circle color intensity corresponds to NES p value with up-regulation in red scale and down-regulation in blue scale. GO terms shown have an NES FDR <0.01 in at least one pairwise comparison. NES values with an FDR >0.01 are not shown. See also Figures S1 and S2.

To characterize what categories of transcripts were differentially expressed between sessions, we performed GSEA on all significant differentially expressed genes for all sampling session comparisons, applying a stringent false discovery rate (FDR) of 0.01. Results for enriched biological processes are shown in Figure 3, and those for molecular functions are shown in Figure S1. There was significant enrichment of GO terms related to RNA translation and protein synthesis (e.g., translation initiation, ribonucleoprotein complex biogenesis, protein localization to endoplasmic reticulum) in the down-regulated set of transcripts, which occurred throughout the protocol compared with baseline and did not show any signs of recovery during R. Indeed, the largest number of down-regulated transcripts was observed in R (compared with BDC1; Figure 2), and these were mostly associated with GO terms related to RNA translation. Large numbers of genes associated with these processes and functions were highly significantly down-regulated (e.g., 144 and 148 ribosomal subunit/mitochondrial ribosomal subunit proteins down-regulated in HDT1 vs. BDC1 and HDT3 vs. BDC1, respectively). The protocol also had immediate and lasting effects on the up-regulation of biological processes related to chemical stimulus detection (Figure 3), and, for example, 28 genes with molecular function related to “olfactory receptor activity” were up-regulated in HDT3 vs. BDC1 (Figure S1).

On entering HDT2, and to a lesser extent during HDT3, there was also large up-regulation in genes with biological processes related to immune and inflammatory function, compared with baseline BDC2. These included “response to type 1 interferon,” “response to interferon-gamma,” “response to interferon-alpha,” “response to interferon-beta,” “neutrophil mediated immunity,” “granulocyte activation,” “regulation of innate immune response,” “leukocyte differentiation,” “cytokine secretion,” and “phagocytosis,” among others. Some of the response to interferon up-regulation showed some down-regulated recovery during R. Thus, through the protocol there was down-regulation of the regulation of gene expression and translation that did not recover, and up-regulation during bed rest only of inflammatory and interferon markers that did show some signs of recovery. Of note, due to the small number of differentially expressed transcripts on transitioning from BDC2 to HDT1, there were no significantly enriched GO terms.

In addition to the up- and down-regulation of GO terms listed earlier, top differentially expressed transcripts were associated with the up-regulation in BDC2, HDT1, HDT3, and R of transcripts coding for many ion channels and receptors (adenosine receptor, 6 calcium channels, cholinergic receptors, chloride voltage-gated channel, dopamine receptor D4, GABA receptor, 4 glutamate receptor subunits, 5 potassium channels, 2 purinergic receptors, and a sodium channel). Of interest, transcripts related to “digestion” were up-regulated in HDT1 compared with BDC2, down-regulated in HDT2 compared with BDC2 and HDT1, and then up-regulated again in R compared with all other sessions. Transcripts associated with the GO term “muscle system process” were up-regulated in R compared with BDC1, BDC2, and HDT1. Transcripts associated with muscle function were also down-regulated during bed rest. In HDT2 the two most down-regulated transcripts compared with HDT1 code for *MYOG*, which is a muscle-specific transcription factor that induces myogenesis and is essential for muscle development. *MYOG* is up-regulated during baseline from BDC1 to BDC2, presumably in response to exercise regimes carried out in this period. It is also the most up-regulated transcript in recovery again probably in response to resumption of regular exercise and locomotion (Figure S2). In addition to *MYOG*, three other myosin-related transcripts (*MYL4*, *MYH14*, *MYBPC2*) were also highly down-regulated in HDT2 and showed expression profiles very similar to *MYOG* throughout the protocol (Figure S2).

### Principal components (PCs) of the blood transcriptome respond differentially to the protocol

**Figure 4:**
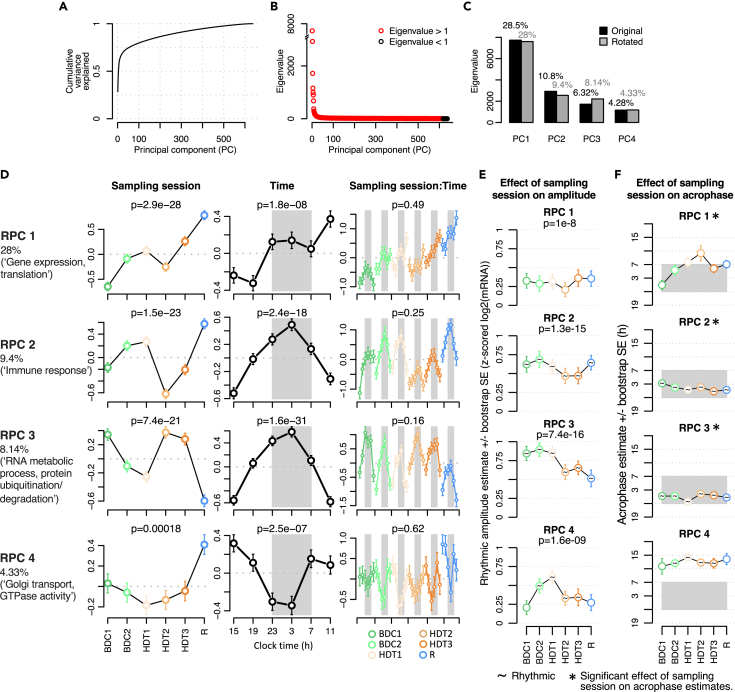
Principal-component analysis (A) Cumulative variance explained across all principal components (PCs). (B) Eigenvalues of all PCs. Red circles identify eigenvalues above 1 and black circles show PC with eigenvalues below 1. (C) Eigenvalues of first four PCs (black bars) and their varimax rotation (gray bars). Percentages correspond to percentage of variance explained. (D) Mixed model ANOVA of rotated principal component (RPC) scores with main effects of sampling session (left column), time (center column), and sampling session:time interaction (right column) for the four RPCs (rows). The significance of these main effects is denoted by their respective p value displayed above each panel. Vertical axes correspond to RPC score least squares mean +/− std. error for sample groups shown on the horizontal axes. Groups in the right column follow a temporal order within each sampling session. (E and F) Rhythmic modeling of RPC score profiles (shown in right column of panel D). Amplitude (E) and acrophase (F) (+/− bootstrap std. error) obtained from rhythmic modeling of scores based on a mixed model with subject-specific random slopes. Effect of sampling session on rhythmic amplitudes was estimated with a mixed model ANOVA; p values of main effect of sampling session are reported above each plot. Effect of sampling session on acrophase values was estimated with a Bayesian circular mixed model; significant changes (95% confidence interval of circular means estimates do not overlap) are indicated with an asterisk above the plots. Lights-off period is indicated by a gray rectangle in panels D and E. See also Figure S3, Tables S1 and S2.

We next performed an unbiased principal-component analysis (PCA) on the gene expression matrix that included all samples from all participants and all sampling sessions. Since the human blood transcriptome is highly individual, and we are primarily interested in changes in the transcriptome, the gene expression matrix was participant centered; i.e., for each transcript and each participant, expression was represented as deviation of the average expression level of that transcript across all samples of that individual. The PCA identified many components that accounted for the variance observed within the data (Figure 4A), and the scree plot suggested that 7 or 8 components with an eigenvalue >1 could be considered (Figure 4B). Here, we consider the first 4 major components (PC1–4) which accounted for 49.9% of the variation in gene expression (rotated principal components [RPCs] 5–8 are presented in Figure S3). A varimax rotation was then performed on the PCs (Figure 4C).

### PCs are defined by specific biological functions

To characterize the biological processes and molecular functions associated with the PCs of the variance in transcript expression, we performed GO enrichment analysis on the top 200 loading transcripts with the highest absolute loading on each RPC (Table S1). Significant GO terms were associated with RPC1, RPC2, and RPC3. Thus, RPC1 and RPC2 can be specifically defined as components associated with gene expression/translation and immune function, respectively, while RPC3 is associated with ribonucleoprotein biogenesis, RNA processing, and mitochondrial gene expression.

To further interpret the RPCs, GO analysis was performed on the top 200 positive and negative contributing transcripts for each RPC (Table S2). For RPC1 no significant GO terms were present for the transcripts with top positive loadings. The transcripts with top negative loadings on RPC1 were associated with terms associated with the regulation of gene expression and translation (e.g., mRNA processing, FDR = 0.000001; RNA splicing, FDR = 0.000008). Similarly, for RPC2 no significant GO terms were associated with the transcripts with positive loadings. The transcripts with top negative loadings on RPC2 were associated mainly with innate immune responses (e.g., granulocyte activation, FDR = < 2.2e-16; neutrophil mediated immunity, FDR = < 2.2e-16). For the RPC3-positive loading transcripts, GO terms were associated with RNA metabolic process (e.g., ribonucleoprotein complex biogenesis, FDR = 0.000004; rRNA metabolic process, FDR = 0.000004). Transcripts with negative loadings on RPC3 were associated with protein ubiquitination and degradation (e.g., ubiquitin protein transferase activity, FDR = 0.01180; proteosomal protein catabolic process, FDR = 0.02626). Finally, transcripts with positive loadings on RPC4 were associated with a mix of terms that included Golgi vesicle transport (FDR = 0.0001) and GTPase activity (FDR = 0.0231). Transcripts with top negative loadings on RPC4 were not associated with significant GO terms. Combined, these results show that most of the variance in expression through the protocol is associated with RNA translation and that associated transcripts were down-regulated through the protocol and more so even in recovery (compared with BDC1).

### PC scores change throughout the protocol and across the 24-h day

The change in the PC scores through the protocol, through the 24-h sampling session (as an average of all sessions), and for both protocol condition and time of day are shown in Figure 4D. All four RPCs varied significantly across the protocol and with time of day. RPC1 (gene expression, translation) and RPC2 (immune response) scores tended to rise from baseline (BDC1 & BDC2) to the start of HDT (HDT1) and then dropped to lower levels at HDT2, which was followed by an increase reaching the highest values during recovery (R). Levels of RPC3 (RNA metabolic process, protein ubiquitination/degradation) decreased from BDC1 to HDT1 and reached high levels during HDT2 and HDT3, followed by a steep decrease during R. RPC4 (Golgi transport, GTPase activity) remained more or less stable until recovery when it reached very high values. Please note the opposite time courses of RPC2 and 3 and that RPC1, 2, and 4 all reached the highest values during recovery.

When averaged across all sampling sessions of the protocol, the diurnal variation differed across the RPCs. RPC1 (gene expression, translation) reached the highest values at the end of the 24 h of sampling, i.e., at 11 a.m., whereas the maxima of RPC2 (immune response) and RPC3 (RNA metabolic process, protein ubiquitination/degradation) were located in the middle of the night, where RPC4 (Golgi transport, GTPase activity) reached its minimum. To investigate the changes in the RPCs across the protocol in more detail, we computed the diurnal variation for each sampling session of the protocol and also computed the acrophase and amplitude of the RPCs. From the sampling session:time panels it can be seen that at baseline all RPCs were rhythmic during the 24 h sampling session, with RPC1-3 peaking at night and RPC4 peaking during the day. However, it can also be seen that the RPC1 acrophase gradually shifted to peak also during the day through the protocol; i.e., two peaks developed. By contrast, the rhythmic profiles of the other RPCs were largely unaffected by the protocol. This was confirmed by the analysis of the acrophase of the RPCs through the protocol which for RPC1 shifted from a night peak toward an early day peak but remained largely unchanged for the other RPCs (Figure 4F). By contrast, the amplitude of RPC1 remained steady through the protocol but amplitudes for the other RPCs varied (Figure 4E). Amplitude of RPC2 showed a distinctive reduction in HDT2, while amplitude of RPC3 showed a steady decline through the protocol, and RPC4 amplitude showed an increase with a dip at HDT2.

Considering together the results from the PC and GO analyses, it could be predicted that two major effects of the protocol would be a shift in the peak of transcripts associated with gene expression and translation from night to day and a significant reduction in the amplitude of immune function transcripts at HDT2.

**Figure 5:**
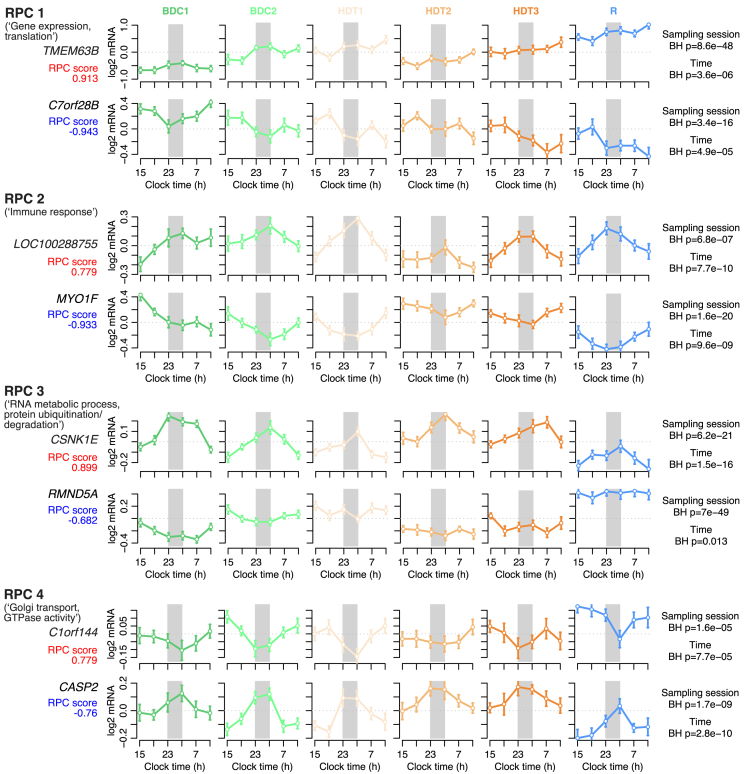
Transcripts with top PCA weighting Average expression profile plots of transcripts with the top rotated principal component (RPC) weighting. Plots show mean average expression (+/− std. error) across participants within each time point. Transcript expression has been zero-centered within participant. Two transcripts per RPC (rows) and across conditions (columns) are displayed for the first four RPCs. Positive RPC scores are shown in red; negative RPC scores are shown in blue. Lights-off period is indicated by a gray rectangle. Right column shows BH-corrected p values from the mixed model ANOVA assessing differential expression among sampling sessions, and time points. See also Figure S4, Tables S1 and S2.

Individual transcripts can contribute positively or negatively to each PC. Figure 5 shows the 24-h temporal expression profiles across the 6 protocol conditions for the top positive and top negative contributing transcripts for each RPC. For each RPC, the positive and negative contributing transcripts have mirror image expression profiles, which broadly agree with the acrophase and amplitude data and overall time course for each RPC in Figure 4. For example, for RPC1, *TMEM63B* (an osmolarity-sensing, calcium-activated ion channel) increased expression levels through the protocol but also became largely arrhythmic. For RPC2 transcripts there is a clear reduction in amplitude at HDT2 for both the top positive and negative transcripts. The top positive-loading transcript in RPC3 is *CSNK1E*, which is a serine/threonine kinase that regulates DNA replication and is also a key component of the circadian molecular clock. Its expression remained largely unchanged through the protocol but showed a distinct reduction in expression during R. In RPC4, the top negative-loading transcript *CASP2* (a caspase that activates stress-induced cell death pathways) showed a similar pattern of expression. Comparison of the top 200 positive- and negative-loading transcripts for each RPC showed that there was almost no overlap (Figure S4).

We also performed a data dimensionality reduction using uniform manifold approximation and projection (UMAP) analysis. This nonlinear approach produced a projection of samples in a two-component space (UMAP1 and UMAP2) that showed a clear separation within that space of samples from the recovery period (Figure S5A). From this type of analysis, it is not possible to identify contributing transcripts, but, overall, the UMAP analysis was consistent with the PCA. UMAP1 and UMAP2 had sampling session and time profiles similar to RPC3 and RPC1, respectively (Figure S5B). The distinct clustering of samples in the two-dimensional space further confirms that the expression of transcripts during recovery was quite different from that during other sampling sessions.

### Effect of the protocol on the number of rhythmically expressed transcripts

It is clear from the RPC analysis that all 4 components represent transcripts with a rhythmic 24-h expression profile and that these profiles are affected by the protocol. Thus, we next sought to characterize the number of transcripts with 24-h rhythmic expression profiles at each sampling session within the protocol. Between individuals, transcripts that are rhythmic can vary in their overall expression level, their rhythmic amplitude and the timing of their peak expression, or acrophase. To account for this, we developed two mixed model regression analysis approaches where a linearized form of a 24-h sine wave was fitted to transcript expression profiles. In one approach, the Y axis intercept was incorporated as a random effect to account for subject-specific variability in baseline expression levels. This allows us to identify rhythmic transcripts that might differ in overall expression levels but still exhibit rhythmic patterns. In the other approach, we introduced random effects for the slopes to allow for variation in rhythmic amplitude and acrophase among participants. In this way, transcripts that are rhythmic but vary with respect to their rhythmic amplitude or timing between individuals can still be detected as rhythmic (see STAR Methods for details). Here we present results for the subject-specific rhythm slopes analysis (Data S2). The distribution of amplitudes and acrophases and the relative number of rhythmic transcripts across sampling sessions are similar in both approaches. The results for subject-specific intercepts analysis are presented as supplemental figures (see in the following).

**Figure 6:**
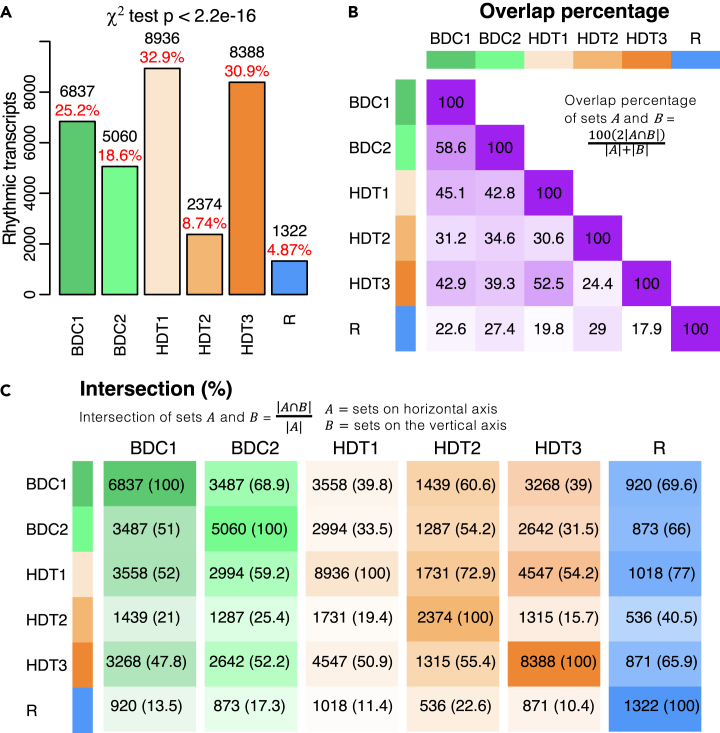
Rhythmic transcripts common across sampling sessions (A) Number and percentage of rhythmic transcripts for each sampling session. Percentage relative to the total number of transcripts analyzed, n = 27,154. The χ^2^ statistic was used to compare the effect of sampling session in the number of rhythmic transcripts. (B) Percentage of overlap of rhythmic transcripts between sampling sessions. Purple color map reflects percentage of overlap with 100% overlap as dark purple and 0% overlap as white. (C) Proportion of rhythmic transcripts in one sampling session that is overlapped by rhythmic transcripts from another sampling session. One color map per column is used with dark color representing 100% and white 0%. All results shown are from rhythmic modeling of transcripts based on a mixed model with subject-specific rhythm slopes. See also Figure S6.

There was a significant effect (p < 2.2e-16, χ^2^ test) of protocol sampling session on the number of rhythmic transcripts (Figure 6A). At the start of baseline, around 25% of all transcripts were rhythmic, which dropped to ∼19% in the second week of baseline. At the start of bed rest there was a large increase to 33%, followed by a large decrease to ∼9% at HDT2. By the end of bed rest this recovered to ∼31%, but this was not maintained in recovery which saw the largest drop to only ∼5% of all transcripts being rhythmic. Throughout the protocol there was a varying percentage of overlapping rhythmic transcripts between pairwise comparisons of session with less overlap with transcripts rhythmic in HDT2 and the least overlap with transcripts in R (Figure 6B). The intersections for the session pairwise comparisons are shown in Figure 6C where columns show the number of rhythmic transcripts that were also rhythmic in the other sessions. This highlights that compared with baseline BDC1 between 48% and 86.5% of transcripts lost rhythmicity through the protocol, with most overlap in HDT1 (52%) and the least in recovery (13.5%). For equivalent results for the random intercepts analysis, see Figure S6. These results show that, although there may be some recovery of rhythmically expressed transcripts during bed rest, this is not maintained in recovery. There is a set of 337 transcripts that remain rhythmic throughout the protocol. Top GO terms enriched within this set included those related to immune function (e.g., interleukin production, T cell activation, cytokine secretion) and included the core clock genes *PER1* and *NR1D1*, and also ghrelin (*GHRL*).

### The protocol affected the expression amplitude and timing of rhythmic transcripts

**Figure 7:**
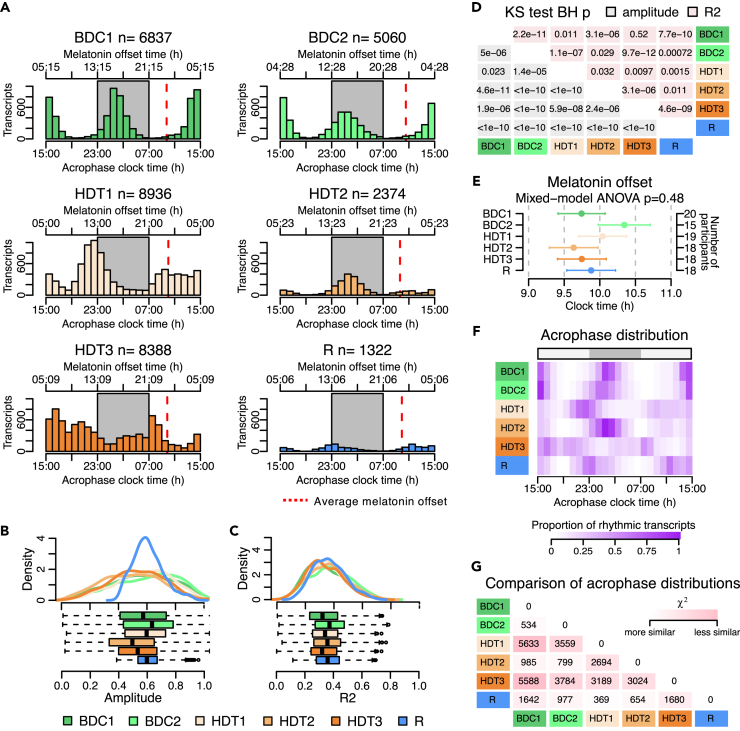
Characterization of rhythmic transcripts (A) Number of rhythmic transcripts (n), acrophase distribution (bottom horizontal and left vertical axes) and average melatonin offset time (top horizontal axis, red dashed line) in the different sampling sessions. Total number of transcripts analyzed is 27,154. Lights-off period is indicated by a gray rectangle. (B) Rhythmic amplitude estimate and (C) model R^2^ distributions of rhythmic transcripts. (D) All pairwise comparisons of amplitude and R^2^ distributions in panels B and C using the Kolmogorov-Smirnov (KS) test. Cells show Benjamini and Hochberg (BH)-corrected p values for amplitude comparisons in gray and R^2^ comparisons in pink. (E) Comparison of melatonin offset times (horizontal axis) across sampling sessions (vertical axis). Plotted values are least squares mean and standard error of fitted values (mixed model ANOVA) for the effect of sampling session. The significance of the sampling session’s effect is indicated by its p value, displayed above the panel. Number of participants in each session is displayed on the right vertical axis. (F) Heatmap of acrophase distribution of rhythmic transcripts per session. Purple-color map indicates the proportion of rhythmic transcripts per session. Lights-off period is indicated by a gray rectangle (top horizontal axis). (G) All pairwise comparisons of acrophase distribution using χ^2^ test. Cells show χ^2^ values. Comparisons are based on the number of transcripts in each 1-hour-acrophase-bin. All χ^2^ have p < 0.05. All results shown are from rhythmic modeling of transcripts based on a mixed model with subject-specific rhythm slopes. See also Figures S7–S10.

Not only the number of rhythmic transcripts but also their amplitude and the 24-h distribution of their acrophases changed through the protocol (Figure 7A). For all transcripts, the distribution of amplitudes and of R^2^ curve-fit values changed through the protocol (Figure S7). For the sets of transcripts that were characterized as rhythmic in this analysis, the distribution of amplitudes (Figure 7B) and of R^2^ curve-fit values (Figure 7C) also changed through the protocol for all session comparisons, although the R^2^ scores for BDC1 and HDT3 were not significantly different (Figure 7D). Amplitudes of rhythmic transcripts were highest at BDC2 and lowest at HDT2. At baseline BDC1 and BDC2 there were 6,837 and 5,060 rhythmic transcripts, respectively, and their temporal distribution of peak expression was biphasic with mainly day- and night-peaking transcripts. On entering HDT1, the number of rhythmic transcripts increased and mainly for the night-peaking transcripts whose peak expression also shifted to an earlier phase. The number of rhythmic transcripts reduced dramatically in HDT2, but their distribution shifted to be more similar to baseline. The number of rhythmic transcripts in HDT3 increased to levels similar to those in HDT1 but with a poorly defined acrophase distribution. Surprisingly, in R the number of rhythmic transcripts fell to the lowest level in the protocol. In this analysis, transcript expression was aligned with melatonin offset, which did not change significantly through the protocol (Figure 7E). Mean melatonin offset appeared to advance in BDC2 compared to BDC1, but in a pairwise comparison this was not significant. Acrophase distributions for all sampling sessions are shown as a heatmap in Figure 7F, and there were significant differences for all pairwise comparisons of the distributions (Figure 7G), with HDT3 being most dissimilar to all other sampling sessions. Taken together, these data reflect the results obtained from the PCA. There are clear changes to the amplitude and acrophase distributions of rhythmic transcripts through the protocol which are similar to changes seen in the RPCs. The equivalent results from the random intercepts analysis were very similar (see Figure S8).

### Biological processes and molecular functions associated with rhythmic transcripts

To understand which categories of rhythmic transcripts were affected by the protocol, we performed GO analysis on subsets of transcripts within specific time windows representing the acrophase peaks for each sampling session in the protocol. At BDC1, the transcripts that peaked during the night (21:00–07:00 h) were associated with GO terms related to translation and the regulation of gene expression (e.g., ribonucleoprotein complex biogenesis, FDR = 2.5135e-13; posttranscriptional regulation of gene expression, FDR = 0.00025286), while the day-peaking transcripts (11:00–18:00 h) were associated with terms related to innate immune responses (e.g., granulocyte activation, FDR <2.2e-16; neutrophil mediated immunity, FDR <2.2e-16). This confirms previous findings for biphasic rhythmic human transcripts.^6^ Similar results were found for the rhythmic transcripts in BDC2.

In HDT1, the night-peaking transcripts whose distribution had shifted earlier (19:00–03:00 h) were still associated with the regulation of gene expression and translation (ribonucleoprotein complex biogenesis, FDR <2.2e-16; translation initiation, FDR = 4.1892e-14), and day-peaking transcripts were associated with immune function (granulocyte activation, FDR <2.2e-16; interleukin-1 production, FDR = 0.00076). This is consistent with the acrophase shift observed for RPC1 which was enriched for transcripts associated with gene expression and translation. HDT1 had the largest number of rhythmic transcripts throughout the protocol. Analysis showed that the most significant GO terms for transcripts that were only rhythmic in HDT1 were associated with RNA translation. Thus, paradoxically, while transcripts associated with RNA translation were down-regulated during HDT1 compared with BDC1 and BDC2, RNA translation-related transcripts also became rhythmic compared with baseline.

At HDT2 night-peaking transcripts were once more aligned with the baseline night-peaking transcripts and were associated with gene expression and translation (e.g., ribonucleoprotein complex biogenesis, FDR = 0.0002), but top GO terms now also included immune-related functions (e.g., leukocyte differentiation, FDR = 0.0084; T cell activation, FDR = 0.046). This could indicate that the reduced number of night-peaking transcripts has revealed immune-related rhythmic transcripts that were previously masked by more numerous gene expression-related transcripts, or it could indicate a reduction in the biphasic temporal organization of these transcripts. At HDT2, day-peaking transcripts were still associated with immune functions, but the top GO terms were now dominated by terms related to interleukin activity (e.g., interleukin-6 production, FDR = 0.00002; interleukin-1 production, FDR = 0.000114; interleukin-12 production, FDR = 0.00075). This change in GO terms associated with immune function may reflect the reduction in the number of rhythmic transcripts observed at HDT2 and also the reduction in rhythmic transcript amplitude, which agrees with the reduced amplitude for transcripts associated with RPC2, which were also enriched for immune function GO terms.

At HDT3, biphasic subsets of rhythmic transcripts were less defined but GO analyses were performed on transcripts peaking between 15:00–23:00 h, and 02:00–10:00 h. Surprisingly, the day-peaking subset was now dominated by terms related to gene expression and translation (e.g., ribonucleoprotein complex biogenesis, FDR <2.2e-16; translation initiation, FDR = 1.6757e-13; translation elongation, FDR = 3.5692e-12). Transcripts that peaked mainly during the night associated with a mix of terms (e.g., transcription coactivator activity, FDR = 0.00036; cell-cell signaling by wnt, FDR = 0.00036; positive regulation of catabolic process, FDR = 0.00036). This indicates that the temporal organization of processes related to gene expression and translation that normally peak during the night have become disrupted and shifted to the daytime.

At R, day-peaking transcripts were again associated with immune function, including interleukin activity (e.g., granulocyte activation, FDR = 5.4544e-9; interleukin-1 production, FDR = 0.000009; interleukin-6 production, FDR = 0.00017). Because of the lowest number of rhythmic transcripts at R, those that peaked mainly during the night (20:00–03:00) did not have associated GO terms with an FDR <0.05, but top terms with a significant p value were again related to a mix of gene expression and translation (e.g., transcription cofactor binding, p = 0.00126) and immune function (e.g., T cell activation, p = 0.00152).

We also performed a weighted gene correlation network analysis (WGCNA) on the gene expression matrix (see STAR Methods). WGCNA involves grouping genes into modules based on their correlated expression patterns. Each module is characterized by its eigengene, which provides a representation of the expression patterns of all the genes in the module. This analysis identified co-expression modules in each sampling session ranging from 22 in R to 35 in HDT3 (see Data S3). Consistent with previous findings in baboons,^21^ many were classified as rhythmic ranging from 7 in R to 22 in HDT3 (Data S3). These rhythmic modules were further classified as representing mainly day or night peaking, but also some morning- and evening-peaking modules presumably associated with the phase shifts in rhythmic peak expression observed during the protocol. Day-peaking and morning-peaking modules were associated with the same categories of immune-related GO terms already identified (Figures S9 and S10, respectively), while night- and evening-peaking rhythmic modules were associated with GO terms related to RNA metabolism/processing and translation (Figures S9 and S10).

### Effect of the protocol on the timing and amplitude of expression of circadian clock genes

**Figure 8:**
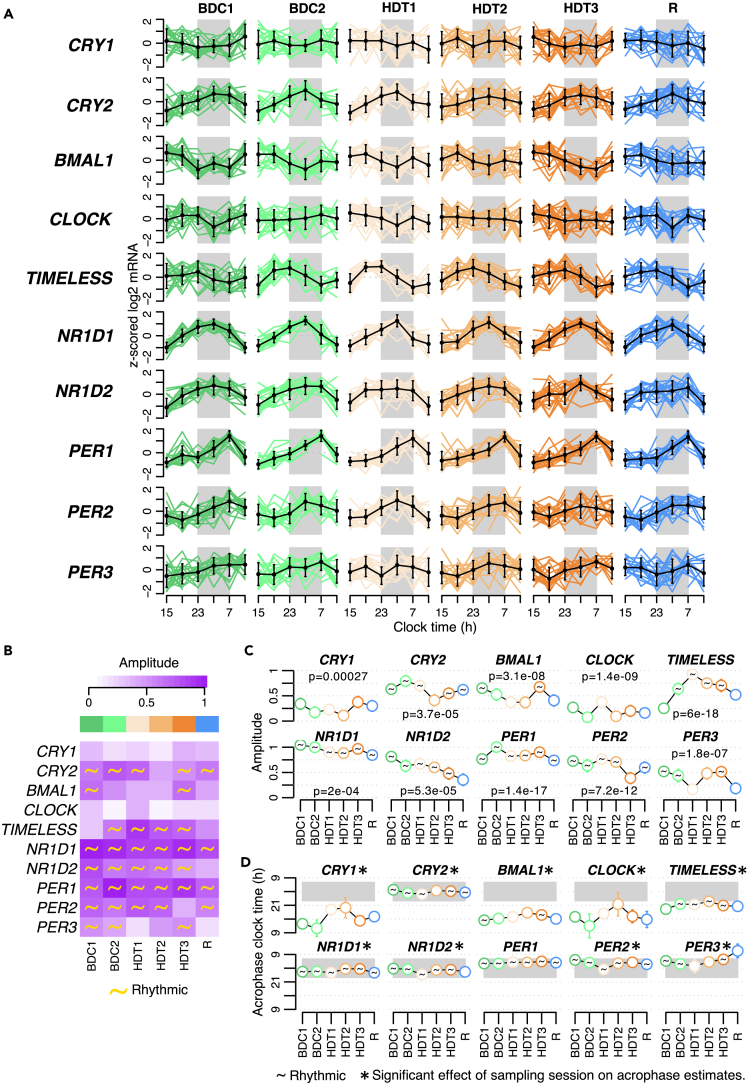
Effect of the protocol on core clock genes (A) Average (mean +/− std. deviation) of *Z*-scored (across time) profiles per sampling session in black and individual *Z*-scored profiles in color. (B) Heatmap of rhythmic amplitude values. A yellow sine wave indicates the transcript is classed as rhythmic in that session. (C) Amplitude values +/− bootstrap std. error per session. Effect of sampling session on rhythmic amplitudes was estimated with a mixed model ANOVA; p values of main effect of sampling session are reported above each plot. (D) Acrophase values +/− bootstrap std. error per session. Effect of sampling session on acrophase values was estimated with a Bayesian circular mixed model; significant changes (95% confidence interval of circular means estimates do not overlap) are indicated with an asterisk above the plots. In panels C and D, a black wave indicates the transcript is classed as rhythmic in that session. All results shown are from rhythmic modeling of transcripts based on a mixed model with subject-specific rhythm slopes. In panels A and D, the lights-off period is indicated by a gray rectangle.

Core clock genes are driving rhythmicity in expression of many genes. In view of the marked effect of the protocol on rhythmicity of the transcriptome, we were interested to determine the effect of the protocol on the set of core clock genes. We characterized (using random slopes analysis) the rhythmicity, acrophase, and amplitude of ten core clock genes throughout the protocol (Figure 8). Individual *Z*-scored expression plots and their mean curves are shown in Figure 8A. The amplitude and rhythmicity of the clock genes varied throughout the protocol (Figure 8B). *NR1D1* and *PER1* were rhythmic throughout the protocol (Figure 8B) and overall had the largest amplitudes (Figure 8C). *CRY2* and *PER2* were also strongly rhythmic but lost rhythmicity during HDT2 and HDT3, respectively (Figure 8B). *CRY1* and *CLOCK* had the lowest amplitudes overall (Figure 8C) and were arrhythmic throughout the protocol (Figure 8B). Of interest, *TIMELESS* was arrhythmic at the start baseline but then became strongly rhythmic with increasing amplitude through the remaining baseline period and on entering HDT, followed by declining amplitude through HDT and R where it again became arrhythmic. Apart from *CRY1*, *CLOCK*, and *TIMELESS*, all other clock genes were rhythmic at baseline (BDC1) and also had high rhythmic amplitudes (Figures 8B and 8C). Six out of ten clock genes were arrhythmic in recovery (R) (Figure 8B). The rhythmic amplitude of *NR1D1* expression remained high throughout the protocol with some decline toward the end, whereas the amplitude of *NR1D2* declined more rapidly with no recovery in R where rhythmicity was also lost (Figure 8C). The acrophases for most of the negative feedback limb genetic components of the molecular clock peaked during the night (*NR1D1* & *NR1D2* early night; *PER1-3* late night; and *CRY2* middle of the night) and remained largely unchanged through the protocol (even though for all clock genes except *PER1* there was a significant effect of sampling session on acrophase). The acrophase of *PER3* during R shifted to the early morning, but expression was no longer classified as rhythmic (Figure 8D). The acrophase for *TIMELESS* through the protocol occurred earlier than the negative limb transcripts peaking just before the dark phase. The acrophase of the positive loop transcription factor *BMAL1* also remained largely unchanged and peaked as expected in antiphase to the negative components. Interestingly, the acrophase for *CRY1* and *CLOCK* changed a lot through the protocol and in a very similar way to each other. However, this may reflect the fact that these transcripts had the lowest amplitudes of all clock genes and were also arrhythmic (Figure 8B).

## Discussion

Many studies have investigated the effects of simulated or actual microgravity on the transcriptome, but of these most are based upon single or few time points. This is the first long-duration bed rest HDT study to fully characterize the effects of bed rest on the temporal 24 h organization of the human whole-blood transcriptome in 24-h time series analyses at six sampling sessions throughout the 90-day protocol. We find that this long-duration intervention has the most profound disruption to the human transcriptome so far recorded.

### Non-monotonic effects of the protocol observed at all levels of transcript expression

Transcript expression changed in a non-monotonic way throughout the protocol. Unbiased PCA identified four PCs accounting for almost 50% of the variance in transcript expression. PCA scores for each component were rhythmic through the protocol and across 24-h sampling sessions with changes in both amplitude and peak timing. The top two components accounted for 37.4% of the variance and were associated with RNA translation and immune function. The translation-enriched PC moved from night peaking toward day peaking through the protocol, whereas the amplitude of score for the immune-enriched component reduced during bed rest HDT. This unbiased approach shows that the protocol had major disruption to translation and immune function transcripts.

### The protocol had immediate and lasting effects on the transcriptome

Even during baseline 38% of transcripts were differentially expressed when comparing BDC1 with BDC2, indicating that simply remaining indoors with reduced exposure to external light has an impact on the transcriptome. Thirty-nine percent of transcripts were up- or down-regulated in the middle of bed rest HDT compared to the start. The largest number of differentially expressed transcripts (76% compared with BDC1) was observed during recovery showing that even by the end of the protocol the temporal organization of the transcriptome did not resemble its original baseline profile. Thus, the protocol had both acute and long-lasting effects on the transcriptome, much of which did not recover.

### Rhythmic transcripts changed in number, amplitude, and phase through the protocol

18%–25% of transcripts were rhythmic during baseline with a characteristic day- and night-peaking distribution confirming our previous findings.^6^^,^^7^ The number of rhythmic transcripts increased substantially on entering bed rest HDT, but the distribution of the night-peaking transcripts shifted earlier toward the end of the day. The middle of bed rest HDT saw a large decrease in the number of rhythmic transcripts, which recovered toward the end of bed rest HDT but with an almost complete loss of the normal day- and night-peaking distribution. The lowest number of rhythmic transcripts (4.8%) was present at the end of recovery meaning that the normal baseline temporal distribution of rhythmic transcripts was not restored. Amplitudes of rhythmic transcripts were lowest at HDT2 and recovery. Seven out of 10 core clock genes were rhythmic at BDC1, but only 4 remained rhythmic in recovery, and the amplitude of most clock gene reduced through the protocol.

### Comparison with our previous transcriptome studies

We have previously shown that acute and chronic sleep loss^7^ and mistimed sleep^6^ disrupted the temporal organization of the human whole-blood transcriptome. We showed that one week of sleep restriction (6 h sleep opportunity) resulted in a 7-fold increase in up- and (mainly) down-regulated transcripts, the latter including transcripts related to gene expression and translation. Sleep restriction also resulted in a decrease in the number of day-peaking rhythmic transcripts. It should be noted that in the current study average nocturnal sleep duration (assessed with polysomnography; Bonmati Carrion et al., under review) was never more than 6.10 h throughout the protocol meaning participants were sleep deprived. Consistent with our previous sleep restriction study, we also found down-regulation of transcripts related to gene expression and translation. However, in the present study we have observed much larger effects on the numbers of differentially expressed transcripts and the temporal organization of rhythmic transcripts. But this long-duration, 90-day bed rest HDT protocol is complex; participants were confined indoors with reduced exposure to outside light, and constant bed rest with HDT resulting in a redistribution of body fluid, much reduced locomotor activity, and constant posture removed an important diurnal feedback signal. The large number of differentially expressed transcripts observed even during baseline when participants were free-moving implies that just being confined to indoors without exposure to high levels of external light impacts the temporal organization of the transcriptome.

We previously showed that mistimed sleep significantly reduced the number of both day- and night-peaking rhythmic transcripts.^6^ Here, the bed rest protocol also had a major disruption to the temporal organization of day- and night-peaking transcripts, with reductions in numbers through the protocol and also a redistribution of peak times, and these effects were not restored in recovery. This implies that circadian timing signals are reduced during bed rest. Consistent with this, the amplitude of expression of most of the core clock genes was reduced through the protocol, with the majority being arrhythmic by the end, although the phase of peak expression of most clock genes remained largely unchanged, except for *CLOCK* and *CRY1* whose phase shifted later. In the previous mistimed sleep study, we observed transcripts that became rhythmic when sleep was mistimed 12 h from the internal biological clock. We showed that this could be modeled as due to the removal of a negative masking effect of sleep on rhythmic transcripts that peak during the biological night. Here, a similar removal of a negative masking effect of the posture cycle might contribute to the initial increase in the number of rhythmic transcripts observed at the start of HDT.

### Biological processes and molecular functions affected by the protocol

Some biological processes and molecular functions affected by the protocol were predictable, but others were more surprising. Consistent with other bed rest studies^14^ we saw down-regulation with some of the largest fold changes of transcripts related to muscle system process (e.g., *MYOG*, *MYL4*, *MYH14*, *MYBPC2*), although expression levels were restored in recovery, and it should also be noted that expression levels of these muscle-related transcripts were generally higher through the protocol compared with the first baseline session. The protocol had immediate and lasting effects, with no recovery, on the down-regulation of transcripts related to protein translation. Mitochondrial gene expression transcripts were also down-regulated, and mitochondrial dysfunction has been observed in other bed rest studies^13^^,^^14^ and also during spaceflight.^22^ Some of the strongest up-regulation was associated with interferon signaling, which has also been recorded in previous bed rest studies.^23^^,^^24^ The National Aeronautics and Space Administration (NASA) twins study, which compared various omics datasets between a ground-based twin and an identical twin on the International Space Station (ISS),^25^ found changes to many immune function pathways during spaceflight including changes to neutrophil activation and granulation, which we also found here. Importantly, another study utilizing the same participants reported here investigated the leukocyte transcriptome in single-time-point samples collected at 10 sessions through the bed rest HDT protocol. The authors report the differential expression of 2,415 protein-coding transcripts that fell into six clusters which were enriched for GO terms related to immune function, sequestration of ions, cellular stress, and mineralization.^26^ Of interest, similar to our results, they also found the largest differences in transcript expression on entering HDT and during recovery. Spaceflight has also been shown to up-regulate olfactory processes,^22^ and another large and immediate effect of the current protocol was the up-regulation of transcripts related to the sensory perception of chemical stimulus, which included transcripts coding for many olfactory receptors. Surprisingly, we also observed the up-regulation of several opsin gene transcripts (*OPN1SW*, *OPN1MW*, *OPN1LW*, *OPN4*) and the down-regulation of *OPN3*. While it may be surprising to detect the differential expression of these transcripts in human blood, we have previously reported the up-regulation of *OPN1LW* in response to chronic sleep loss.^7^

### Conclusion and outlook

This study created the longest-duration time series human transcriptomic dataset so far available. The dataset represents a resource which can be used for further analyses of the dynamics of the transcriptome, trait-like characteristics of the transcriptome and its response, and testing of published biomarkers for circadian phase.^27^^,^^28^^,^^29^^,^^30^ The results of the analyses presented here show that the 90-day bed rest protocol uncovered dynamic, non-monotonic changes to amplitude, phase, and temporal organization of the human whole-blood transcriptome and by far the largest disruption to temporal gene expression compared with existing data. How these large transcriptome changes relate to changes in the temporal organization of the proteome and metabolome, both of which are under circadian control and affected by, for example, sleep deprivation or mistimed sleep^31^^,^^32^^,^^33^^,^^34^ remains to be investigated. The findings we present here already have important implications for understanding the effects of spaceflight, but also for our understanding of the effects of situations where individuals are confined to long-duration bed rest, such as in hospitals and care settings.

### Limitations of the study

In this study, we used the gold-standard PAXgene RNA collection system that samples from whole blood. This approach greatly improves consistency and quality of RNA samples. One potential criticism of this approach is that in our time series data across multiple sampling sessions, we do not know the relative transcriptomic contributions of different blood cell types. However, this bed rest HDT project included multiple teams investigating different scientific areas and one team studied the total cell counts of blood cells at sampling sessions very close to ours. They found no significant effect of the protocol in cell counts of white blood cells, total lymphocytes, B cells, and B cell subsets.^35^ Thus, our view is that this method provides a time-stamp overview of gene expression in the whole system in response to a challenge and that the observed variation is not primarily driven by variation in fluctuating blood cell numbers. In this study, we utilized microarray technology which has been widely used and optimized over the past few decades, resulting in the availability of a wealth of protocols, databases, and analysis tools, thus establishing it as a reliable and accessible technique. While microarrays are a valuable tool for gene expression analysis, RNA sequencing (RNA-seq) offers a wider dynamic range and is not susceptible to the biases associated with probe design. In contrast to microarrays, which can only profile predetermined transcripts, RNA-seq enables comprehensive sequencing of the entire transcriptome. This study only included male participants, and therefore the results cannot be directly interpreted in relation to females. We acknowledge this but note that this was a consequence of specific aspects of recruitment criteria that we could not influence. This study included an antioxidant “cocktail” intervention that was administered daily to 10 participants during the HDT phase of the protocol (see STAR Methods). Our results showed no effect of this intervention on differential transcript expression, and Stratis et al.^26^ also reported no effect on differential gene expression in leukocytes in the same participants. Three other studies with the same participants also showed no effect of the intervention on bone density/structure,^36^ bone resorption/formation,^37^ and muscle deconditioning.^38^ By contrast, one study showed a positive effect on lipid and oxidative stress homeostasis,^39^ while another showed the intervention attenuated antioxidant stress responses and muscle deterioration.^40^ Finally, we also acknowledge that this study represents a single omics approach, and we cannot say anything about temporal changes that may have occurred at the level of the proteome, although temporal changes in gene expression will directly influence the dynamics of the proteome. As in any clinical study, there were strict limits on the total amount of blood that could be drawn from each participant. Because the overall study involved many cooperating research teams many of which required blood samples, we were not able to take additional blood samples to study other omic features.

## STAR★Methods

### Key resources table

**Table:** 

REAGENT or RESOURCE	SOURCE	IDENTIFIER
**Deposited data**		

Raw and pre-processed data	This paper	GEO: GSE253864

**Software and algorithms**		

R (v 4.2.2)	https://www.R-project.org/	https://www.R-project.org/
limma R package (v3.46.0)	Ritchie et al.^41^ https://doi.org/10.1093/nar/gkv007	https://bioconductor.org/
sva R package (v3.38.0)	Leek et al.^42^ https://doi.org/10.1093/bioinformatics/bts034	https://bioconductor.org/
biomaRt R package (v2.54.1)	Durinck et al.^43^ https://doi.org/10.1093/bioinformatics/bti525	https://bioconductor.org/
Seurat R package (v4.3.0.1)	Hao et al.^44^ https://doi.org/10.1016/j.cell.2021.04.048	https://satijalab.org/seurat/
lmerTest R package (v3.1-3)	Kuznetsova et al.^45^https://doi.org/10.18637/jss.v082.i13	https://github.com/runehaubo/lmerTestR
umap R package (v0.2.10.0)	McInnes et al.^46^https://doi.org/10.48550/arXiv.1802.03426	https://github.com/tkonopka/umap
MuMIn R package (v1.42.17)	Burnham and Anderson^47^https://doi.org/10.1007/978-1-4757-2917-7	https://github.com/cran/MuMIn
bpnreg R package (v2.0.2)	Cremers et al.^48^https://doi.org/10.1111/bmsp.12108 Nuñez-Antonio and Gutiérrez-Peña^49^https://doi.org/10.1177/1471082X100110	https://github.com/joliencremers/bpnreg
Webgestalt	Zhang et al.^50^ https://doi.org/10.1093/nar/gki475	https://www.webgestalt.org/
WebGestaltR R package (v0.4.4)	Zhang et al.^50^ https://doi.org/10.1093/nar/gki475	https://github.com/bzhanglab/WebGestaltR
WGCNA R package (v1.72-1)	Langfelder and Horvath^51^ https://doi.org/10.1186/1471-2105-9-559	https://github.com/cran/WGCNA

### Resource availability

#### Lead contact

Further information should be directed to and will be fulfilled by the lead contact, Simon Archer (simon.archer@surrey.ac.uk).

#### Materials availability

This study did not generate new unique reagents.

#### Data and code availability

• Microarray data have been deposited at GEO and are publicly available as of the date of publication. Accession numbers are listed in the key resources table.
• This paper does not report original code.
• Any additional information required to reanalyse the data reported in this paper is available from the lead contact upon request.

### Experimental model and study participant details

#### Study protocol

Twenty healthy male volunteers (age 20-45, 34.15 ± 7.63, mean ± SD) participated in the study, which was approved by the Comité de Protection des Personnes SUD-OUEST ET OUTRE-MER I, and by the Agence nationale de sécurité du medicament et des produits de santé (ClinicalTrials.gov database number NCT03594799). All participants were French with ethnicity recorded as Black African origin n = 1, African Caribbean origin n = 1, Latin American origin n = 1, White n = 17. Written informed consent was obtained from each participant prior to the study. Recruited participants completed a medical questionnaire and a clinical assessment and did not suffer from a sleep disorder nor perform shift work, nor travelled across more than 1 time zone in the 2 months prior to starting the study. The study was conducted at the MEDES (Institute for Space Medicine and Physiology, Toulouse, France) Clinical Unit and was divided in two legs, the first occurring January-April 2017, and the second in September-December 2017. Each of these legs enrolled 10 volunteers. During the study, sleep periods were scheduled from 23:00 (lights off) to 07:00 (lights on). Participants were exposed to room lights and external light via windows in a temperature-controlled environment (20 – 25 ± 0.5°C; mean (± SEM) daily light levels ranged from 36.85 ± 4.24 lux in HDT3 to 88.02 ± 10.63 lux during BDC1). Meals were schedule to 07:30, 12:30, 19:30, with a snack at 16:00. During bed rest, hygiene tasks were performed in bed and showers were performed in horizontal cradles. For each leg, the group was randomly divided into ‘Control’ (5+5 participants) and ‘Cocktail’ (5+5 participants) subgroups. ‘Cocktail’ participants took a combination of anti-inflammatory compound pills that included polyphenols, vitamin E, and omega 3. During the initial 2 weeks of baseline, participants performed a protocol of treadmill or bicycle exercises for 20 to 30 minutes and soft stretching exercises to avoid cardiovascular deconditioning before entering the constant bed rest period. To recover fitness before discharge from the research facility, the participants performed exercise for 20 to 30 minutes daily during the recovery period consisting of walking, treadmill, bicycle, stretching, adapted to the cardiovascular and muscular status of each participant. Resident doctors and nurses attended to participants and assisted with sample collection and routine assessments. Please note that few transcripts (3,347) were differentially expressed when comparing results from each leg, and no transcripts were differentially expressed when comparing cocktail and control groups (see Mixed Model methods and Data S2) and these were not considered further here.

### Method details

#### Sample collection and microarray protocol

2.5ml of whole blood was collected via an indwelling cannula into PAXGene RNA tubes (BD Biosciences). RNA was extracted and checked for concentration and purity as previously described (Moller-Levet 2013). RNA was labelled and hybridised to Agilent human whole genome microarrays (SurePrint G3 8x60k), as previously described.^7^

#### Pre-processing of microarray data

Microarray samples were retained if they had a median coefficient of variation of less than 10% in non-control replicated probes (variable ‘'Metric_gNonCntrlMedCVProcSignal'’ in Feature Extraction (FE) files), had less that 35% of non-control 44k probes (all probes in microarray with GEO accession no. GPL15331) flagged in any of the FE QC variables (variables used: gIsSaturated, gIsFeatNonUnifOL, gIsFeatPopnOL, gIsPosAndSignif, gIsBGNonUnifOL or gIsBGPopnOL), and had a RIN larger than 6. Out of 701 samples, 637 passed the filtering criteria. Log 2 values were quantile-normalized using the normalizeBetweenArrays function from R package limma (v3.46.0). Non-control 44k probes were extracted resulting in a Gene Expression Matrix (GEM) of 41,619 transcripts in 637 samples. The GEM was batch corrected for leg using the ComBat function from R package sva (v3.38.0). Transcripts flagged in any of the FE QC variables in less than 20% of the samples were retained for all analyses (n=27,154).

### Quantification and statistical analysis

#### Melatonin offset values

For the transcript expression analyses presented here, we aligned all rhythmic transcript expression profiles with individual mean melatonin offset times. For melatonin measurement, saliva samples were collected hourly from 7:00 to 23:00. Melatonin concentrations were quantified by radioimmunoassay (Stockgrand Ltd., University of Surrey, Guildford, UK), with a detection limit of 0.85 pg/ml. The intra assay coefficients of variation (CV) for the low (mean ± SD, 6.3 ± 1.0 pg/ml), medium (29.3 ± 3.7 pg/ml) and high (58.7 ± 6.3 pg/ml) pools were 15.9, 12.5 and 10.7 %, respectively. All samples collected for each participant were measured in duplicate in a single assay. The phase marker for melatonin offset was calculated as described by Voultsios et al.,^52^ i.e., determining a threshold from the mean plus two standard deviations of three baseline samples. For melatonin offset, the calculation was determined as the time when melatonin went below that threshold. The effect of sampling session on melatonin offset times was estimated using a mixed model ANOVA with the lmer function from the lmerTest R package (v 3.1-3)). In the model, sampling session (BDC1, BDC2, HDT1, HDT2, HDT3, R) was specified as the fixed effect, while participants were included as random effects. The model was defined as follows: ‘Melatonin offset ∼ Sampling session + (1|Participants)’. We employed a mixed model ANOVA to compare melatonin offset times across sampling sessions because these times were determined from the same participants during various sampling sessions. Our goal was to understand how these repeated measurements changed over time while accounting for individual differences. Least squares means and standard error were produced with ls_means function from lmerTest R package.

#### Mixed model ANOVA of transcript expression levels

Analyses were performed on quantile normalised data with no batch correction for leg. Mixed model ANOVA of each transcript expression was performed using the lmer function from the lmerTest R package (v 3.1-3). The model’s fixed effects included sampling session (BDC1, BDC2, HDT1, HDT2, HDT3, R), sampling time point (15:00, 19:00, 23:00, 03:00, 07:00, 11:00) treated as a categorical variable, group (control vs. cocktail), leg (January – April vs. September – December) and interaction of sampling session and sampling time point, while participants were defined as random effects. The model was specified as follows: ‘Expression level ∼ Sampling session + Time point + Group + Leg + Sampling session:Time point + (1|Participants)’. We employed a mixed model ANOVA to compare transcript expression levels across sampling sessions and time points. This approach allows us to understand how the repeated measurements change over time while accounting for individual differences. Additionally, we were interested in the effects of time point, group, leg, and the interaction of sampling sessions and time point on gene expression, so these variables were added to the model. Least squares (LS) means and standard error, and pairwise differences of LS means for all factors in the linear mixed model were produced with ls_means and difflsmeans functions from lmerTest R package. P-values of main effects and interaction were corrected for multiple testing across all transcripts. P-values of all pairwise contrasts of the main effect of sampling session were corrected for multiple testing within each transcript. Benjamini and Hochberg (BH) method^53^ was used for multiplicity correction in all instances. Transcripts were classed as having a significant difference in expression level if they had a BH p < 0.01 for main effect of sampling session, and a BH p < 0.01 in the selected contrast.

#### Principal component analysis

The batch corrected GEM consisting of 27,154 transcripts in 637 samples) was used for the Principal Component Analysis (PCA). Each transcript was zero-centred within participant. On average, there were 31.9 samples per participant (standard deviation = 2.6). The PCA was performed using the prcomp R function. The varimax rotation matrix for the first four Principal Components (PC) was obtained by applying the varimax function in R to the raw loadings. This rotation matrix was then used to calculate the Rotated Principal Component (RPC) scores.

We employed mixed model ANOVA to assess the impact of Sampling session, Time point, and their interaction on the distribution of PC or RPC scores. Mixed model ANOVA was performed on each of the PC or RPC scores using the lmer function from the lmerTest R package (v 3.1-3). In the model, the fixed effects included Sampling session (BDC1, BDC2, HDT1, HDT2, HDT3, R), Time point (15:00, 19:00, 23:00, 03:00, 07:00, 11:00) treated as a categorical variable, and their interaction, while participants were defined as random effects. The model was specified as follows: ‘PC ∼ Sampling session + Time point + Sampling session:Time point + (1|Participants)’. This approach was chosen because we had PC scores from the same participants at different time points and we aimed to understand how repeated measurements changed over time while accounting for individual differences. Least squares means and standard errors were calculated using the ls_means function from the lmerTest R package. Rhythmic amplitude and acrophase of RPC scores were determined using the linear mixed-effects model with subject-specific rhythm slopes as described in the Rhythmic modelling section.

#### Uniform manifold approximation and projection

Uniform Manifold Approximation and Projection (UMAP) from the umap R package (v0.2.10.0) was used to perform dimensionality reduction of the batch-corrected GEM (27,154 transcripts in 637 samples). Similar to the PCA, each transcript was zero-centred within participants prior to the analysis. We conducted mixed model ANOVA on each of the two layout components using the lmer function from the lmerTest R package (v 3.1-3). The fixed effects in the model included Sampling session (BDC1, BDC2, HDT1, HDT2, HDT3, R), Time point (15:00, 19:00, 23:00, 03:00, 07:00, 11:00) treated as a categorical variable, and their interaction, while participants were defined as random effects. The model was specified as follows: ‘Layout component ∼ Sampling session + Time point + Sampling session:Time point + (1|Participants)’. We chose mixed model ANOVA for this analysis because we had layout components from the same participants at different time points and our aim was to understand how repeated measurements changed over time while accounting for individual differences. Least squares means and standard errors were calculated using the ls_means function from the lmerTest R package.

#### Rhythmic modelling

Data collected in this study follows a repeated measurement design, meaning that the same subject is repeatedly measured multiple times over a period of time. While there are several methods for analysing the rhythmicity analysis of the transcriptome, most of them do not take into account the within-subject correlation resulting from these repeated measurements.^54^^,^^55^ In addressing this concern, recent advancements have introduced linear mixed model-based methods.^56^^,^^57^^,^^58^^,^^59^ These approaches offer a promising solution to capture the underlying rhythmic patterns in the data while accounting for the inherent correlation between repeated measurements within each subject.

Here, a curve of the form y=mes+ampsin(2π(t−φ)/τ)*,* where mes is MESOR, amp is amplitude, *t* is time, φ is phase and τ is period, was fitted to each transcript temporal expression profile using a linearized form of the equation defined by $y=mes+\beta_{1}\;\cos\left(2\pi t/\tau \right)+\beta_{2}\;\sin\left(2\pi t/\tau \right)$, where $\beta_{1}=amp\;\cos\left(2\pi t/\tau \right)$, $\beta_{2}=amp\;\sin\left(2\pi t/\tau \right)\text{,}$ and a fixed τ of 24 h. Similar to (23-26), we assed rhythmicity using linear mixed-effects models allowing for subject-specific intercept and rhythm-slopes (i.e., $\beta_{1}$ and $\beta_{2}$, slopes of the cosine and sine terms in the model). The use of subject-specific intercept allows variability in baseline expression levels, while subject-specific rhythm slopes allow for variation in rhythmic amplitude and acrophase among participants. Amplitude and phase values were derived from the model coefficients such that $amp=sqrt\left({\beta_{1}}^{2}+{\beta_{2}}^{2}\right)$ and $\varphi =\left(\tau /2\pi \right)\tan^{-1}\left(\beta_{1}/\beta_{2}\right)$ (+ τ/2 if $\beta_{1}<0$ and + τ if $\beta_{1}>0$ and $\beta_{2}<0$). Acrophase (i.e., peak time) = (τ−φ+1/4)modτ. FDR values (BH corrected p values) of $\beta_{1}$ and $\beta_{2}$ were used to classify the transcripts as rhythmic flowing min(FDR_β1,_ FDR_β2_)<0.05.

The lmer function from the R package lmerTest (v3.1-3) was used to fit two mixed effects models: 1) A mixed-effects model with subject-specific rhythm slopes (zero intercept and random slopes on z-scored data). The model was specified as follows: ‘Expression level ∼ 0 + x1 + x2 + (0 + x1 + x2|Participant)’, where x1= cos(2πt/τ) and x2= sin(2πt/τ)’; 2) A mixed-effects model with subject-specific MESOR (random intercepts on non-standardized data). The model was specified as follows: ‘Expression level ∼ x1 + x2 + (1|Participant), where x1= cos(2πt/τ) and x2= sin(2πt/τ)’.

Bootstrap standard errors for MESOR, amplitude and acrophase were calculated using the boot function from the boot R package with R=200. The circular nature of acrophase values is not considered by the boot function, therefore, we used the sd.circular function from the circular R package to calculate the standard error from the 200 bootstrap acrophase values. The conditional coefficient of determination (R^2^) for generalized mixed-effect models was calculated using the R.squaredGLMM function from package MuMln (v1.42.17). All pairwise comparisons across conditions of rhythmic amplitude and R^2^ distributions were performed using Kolmogorov-Smirnov tests (ks.test function in R). All pairwise comparisons across conditions of acrophase distribution were performed using χ^2^ tests based on the number of transcripts allocated to 24 one-hour-acrophase bins.

Rhythmicity analysis of transcriptomic data was performed on quantile normalised data with no batch correction. Sampling time points were in decimal units and referred to melatonin offset times, such that time = sampling time – melatonin offset time. Time-series where melatonin offset value is missing were not considered.

#### Effect of sampling session on rhythmic parameters

Rhythmic amplitude and acrophase of clock genes, PC scores and RPC scores were determined using the linear mixed-effects model with subject-specific rhythm slopes as described in the Rhythmic modelling section. These models generated estimates of amplitude and acrophase for each participant, for each time point. We used these estimates to investigate the effect of the sampling session.

For amplitude, we performed a mixed model ANOVA with main effect of sampling session (BDC1, BDC2, HDT1, HDT2, HDT3, R), treating participants as random effects. We employed the lmer function from the lmerTest R package. The model was specified as follows: ‘Rhythmic amplitude ∼ Sampling session + (1|Participants)’. The p-values for the main effect of sampling session are reported.

For acrophase we fit a Bayesian circular mixed model ^60^ with main effect of sampling session (BDC1, BDC2, HDT1, HDT2, HDT3, R) and participants as random effects using bpnme function from bpnreg R package (v2.0.2). The model was specified as follows: ‘Acrophase ∼ Sampling session + (1|Participants)’. This approach does not produce a p-value but mean and 95% confidence interval of circular means [95% of the highest posterior density (HPD) in Bayesian statistics]. If intervals of the estimates do not overlap, we can reject the null hypothesis that the circular means (of acrophase values) for the different sampling sessions do not differ.

#### Microarray data biotype distribution

The processed dataset comprised 27,154 Agilent probes. Among these, 24,732 (91.08%) can be mapped to a gene symbol, while 21,583 (79.48%) have a corresponding biotype assignment for a gene symbol. Biotype annotations for the gene symbols or their synonyms were obtained using the biomaRt R package (v 2.54.1) with Human genes (GRCh38.p14). Gene symbol synonyms were obtained with the GeneSymbolThesarus function from the Seurat R package (v 4.3.0.1). We assessed the biotype distribution of transcripts across four categories: protein-coding, long non-coding RNAs (lncRNAs), unannotated transcripts, and a miscellaneous ‘other’ category. More than 95% of the transcripts within the processed dataset mapped to either protein-coding genes (75.4%, n = 20,466), or lacked a biotype annotation (21%, n = 5,571). The remaining transcripts mapped to lncRNAs (1.3%, n = 356), or were in the ‘other’ category (2.8%, n = 761). Across all sampling sessions, there was a significant difference (P < 0.05, χ^2^ test) in the biotype distribution of rhythmic transcripts compared to the background distribution among all transcripts. Notably, the distribution did not significantly differ among BDC1, BDC2, HDT2, and R, nor among HDT1 and HDT3. However, there was a significant difference in distribution between these two sets (see Data S4).

#### Gene ontology and gene set enrichment analysis

Gene ontology (GO) enrichment analysis was performed using Webgestalt 2019 (webgestalt.org) using over-representation analysis with geneontology databases ‘biological process, noredundant’ and ‘molecular function, noredundant’. The reference set used was the ‘agilent wholegenome 4x44k v2’. Please note that while we used the 8x60k Agilent microarrays, for all analyses presented here we extracted the same 44k feature set used in the 4x44k v2 arrays (GEO accession no. GPL15331; see 7 for details). Gene Set Enrichment analyses (GSEA) were performed with the lists of differentially expressed genes from each sampling session pairwise-comparison, considering GO annotations implemented in Webgestalt (noredundant biological process and molecular function datasets), using the R package WebGestaltR (v0.4.4). Input files contain gene symbol and fold change of differentially expressed genes (BH p < 0.01) for each comparison. All terms with FDR <0.01 in any comparison were selected for plotting.

#### Weighted gene correlation network analysis (WGCNA)

The batch-corrected GEM consisting of 27,154 transcripts in 637 samples was used for Weighted Gene Correlation Network Analysis (WGCNA) using the WGCNA R package. Transcripts in the lower 15% of mean expression and coefficient of variation, across all samples, were filtered out leaving 19,008 transcripts. Transcript expression profiles per participant, per sampling session, were z-scored. A WGCNA was generated independently for each sampling session. All samples and transcripts passed the default settings of the goodSamplesGenes function and visual inspection of the clustering dendrogram was used to identify outliers. Construction of the signed gene networks and identification of the modules was performed using the blockwiseModules function (power=10 based on pickSoftThreshold function). The mixed-effects model with subject-specific rhythm slopes approach (see STAR Methods section “Rhythmic modelling”) was used to assess rhythmicity in the module eigengenes. For each sampling session, rhythmic modules were grouped into four classes based on their estimated peak time (day*-*peaking 11:00-18:00, night*-*peaking 21:00-07:00, morning*-*peaking 07:00-11:00, and evening -peaking 18:00-21:00). Over representation analysis was run for the set of transcripts belonging to each of the four module groups using the R package WebGestaltR (v0.4.4) utilising ‘noredundant’ biological process and molecular function GO annotations. The reference set (27,154 transcripts) and input files contained gene symbols. For plotting, only top 40 terms with FDR<0.01 in at least one sampling session are included.

### Additional resources

Further information about this clinical trial can be found at the National Library of Medicine Clinical Trials registry ‘clinicaltrials.gov’, clinical trial registration code NCT03594799.

## Supplemental information

Document S1. Figures S1‒S10 and Tables S1 and S2

Data S1. Differentially expressed transcripts through the protocol, related to Figure 2

Data S2. Rhythmic transcripts through the protocol, related to Figures 6 and 7

Data S3. Weighted gene correlation network analysis results, related to Figures S9 and S10

Data S4. Microarray data biotype distribution, related to STAR Methods
