# Extensive dynamic changes in the human transcriptome and its circadian organization during prolonged bed rest

**DOI:** 10.1016/j.isci.2024.110364

**Published:** 2024-06-28

**Authors:** Simon N. Archer, Carla Möller-Levet, María-Ángeles Bonmatí-Carrión, Emma E. Laing, Derk-Jan Dijk

## Main text

(iScience *27*, 109331; March 15, 2024)

Due to errors introduced during the drafting of the manuscript, the STAR Methods text under the “rhythmic modelling” heading within the “quantification and statistical analysis” section included incorrect mathematical formulae and references. The authors apologize for this oversight. The STAR Methods text has been corrected in the published article.

