# Supplementary material for: Extensive dynamic changes in the human transcriptome and its circadian organization during prolonged bed rest
Source: iScience. 2024 Feb 22;27(3):109331. doi: 10.1016/j.isci.2024.109331 (PMC10937834; doi:10.1016/j.isci.2024.109331)
Supplement: Document S1. Figures S1‒S10 and Tables S1 and S2 [file mmc1.pdf]

iScience, Volume *27*

Supplemental information

Extensive dynamic changes in the human transcriptome and its circadian organization during prolonged bed rest

Simon N. Archer, Carla Möller-Levet, María-Ángeles Bonmatí-Carrión, Emma E. Laing, and Derk-Jan Dijk

Supplemental information

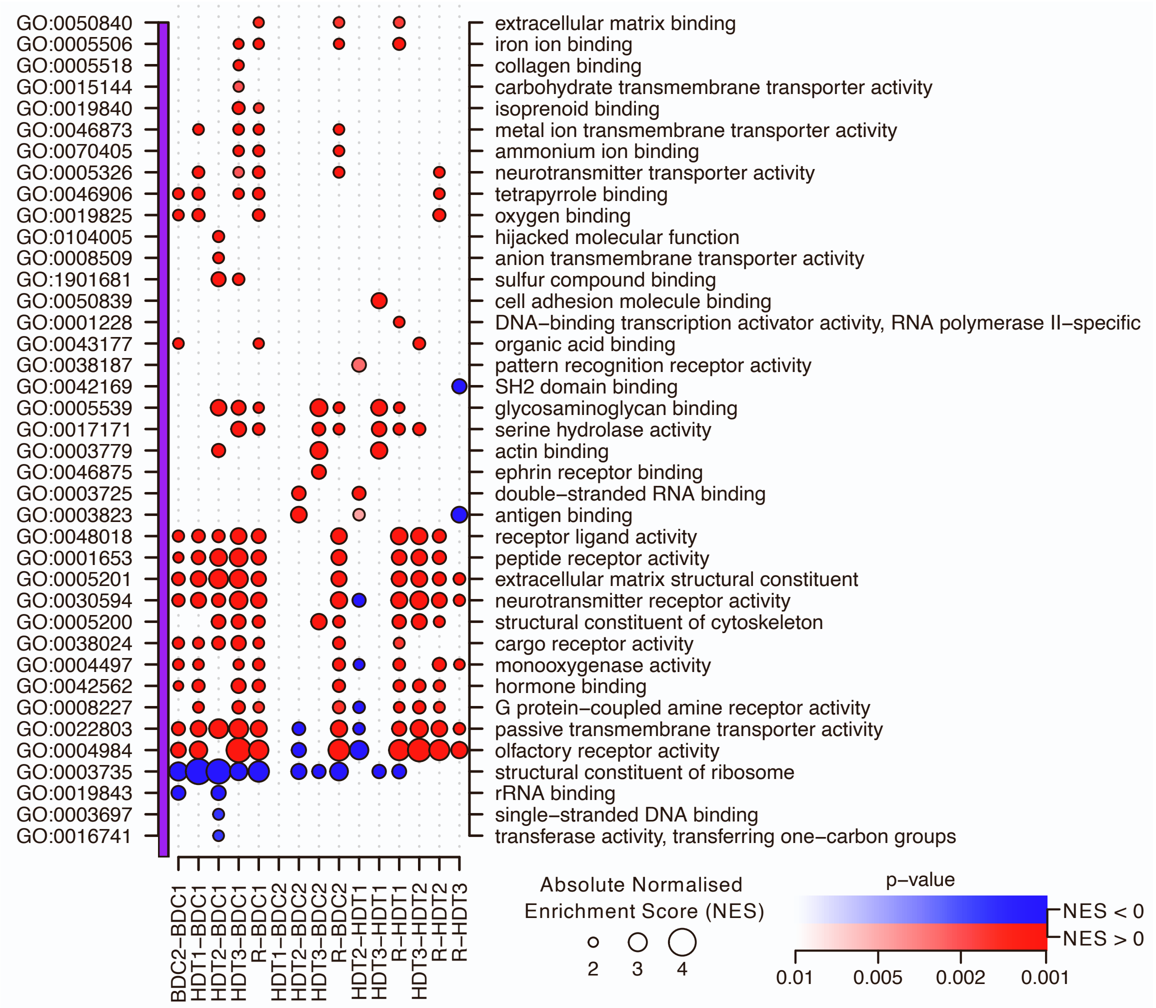

Figure S1. Functional enrichment of differentially expressed genes, Related to Figure 3. Gene Ontology (GO) molecular function enrichment of differentially expressed genes based on gene set enrichment analysis (GSEA). Vertical axes display GO terms (left) and their description (right). Horizontal axis indicates comparison between sampling sessions (e.g., red circle in BDC2-BDC1 indicates up-regulation in BDC2). Circle diameter corresponds to the normalised enrichment score (NES), circle color intensity corresponds to p-value with up-regulation in red scale and down-regulation in blue scale. GO terms shown have a NES FDR < 0.01 in at least one pairwise comparison. NES values with FDR > 0.01 are not shown.

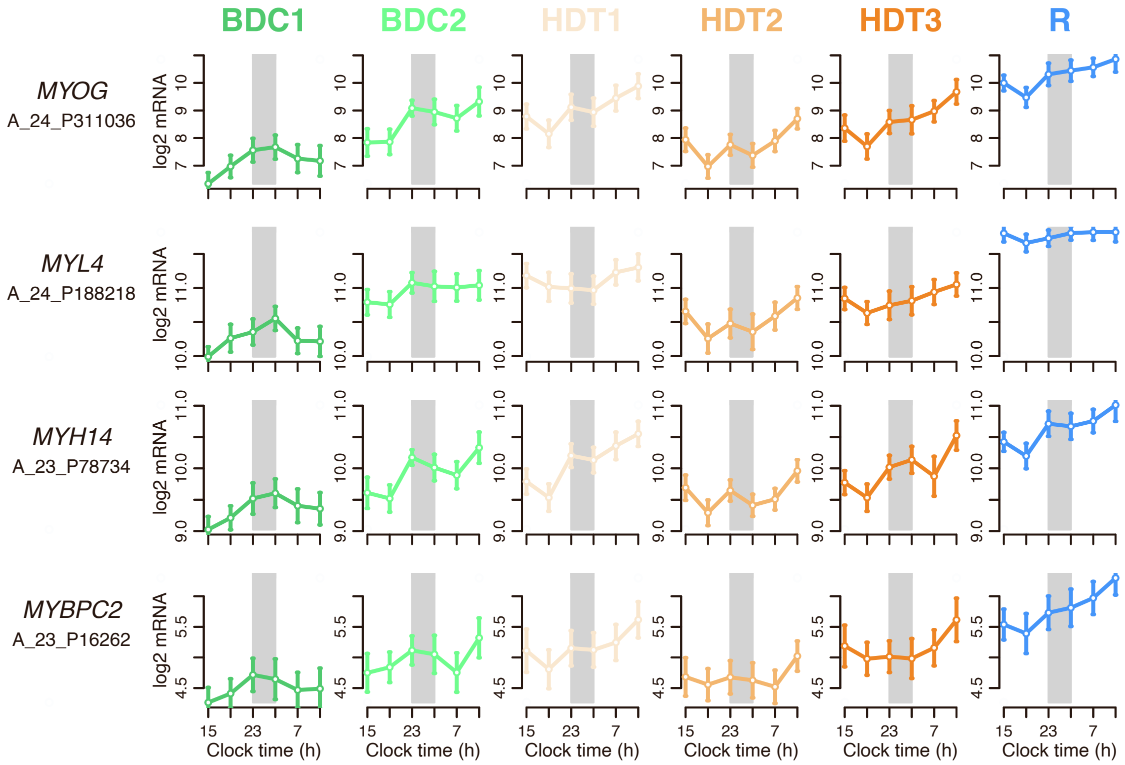

Figure S2. Expression profiles of myosin-related transcripts through the protocol, Related to Figure 3. Plots are mean expression levels +/- SE.

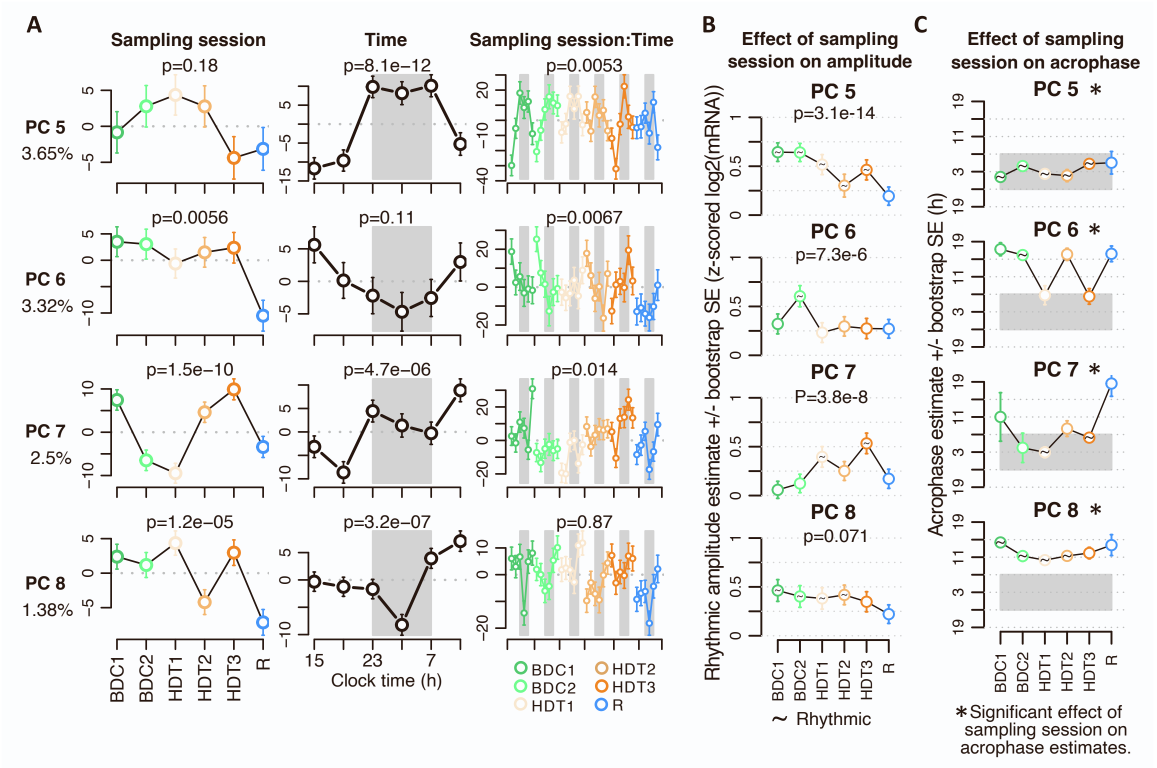

Figure S3. Principal component analysis, Related to Figure 4. A Mixed model ANOVA of principal component (PC) scores with main effects of sampling session (left column), time (centre column) and sampling session:time interaction (right column) for the PC 5 – 8 (rows). Vertical axes correspond to PC score least squares mean +/- std. error for sample groups shown on the horizontal axes. Groups in the right column follow a temporal order within each sampling session. **B**, **C** Rhythmic modelling of PC score profiles. Amplitude (**B**) and acrophase (**C**) (+/- bootstrap std. error) obtained from rhythmic modelling of scores based on a mixed-model with subject-specific rhythm slopes. Effect of sampling session on rhythmic amplitudes was estimated with a mixed model ANOVA; p-values of main effect of sampling session are reported above each plot. Effect of sampling session on acrophase values was estimated with a Bayesian circular mixed model; significant changes (95% confidence interval of circular means estimates do not overlap) are indicated with an asterisk above the plots. Lights-off period is indicated by a grey rectangle in panels **A** and **C**.

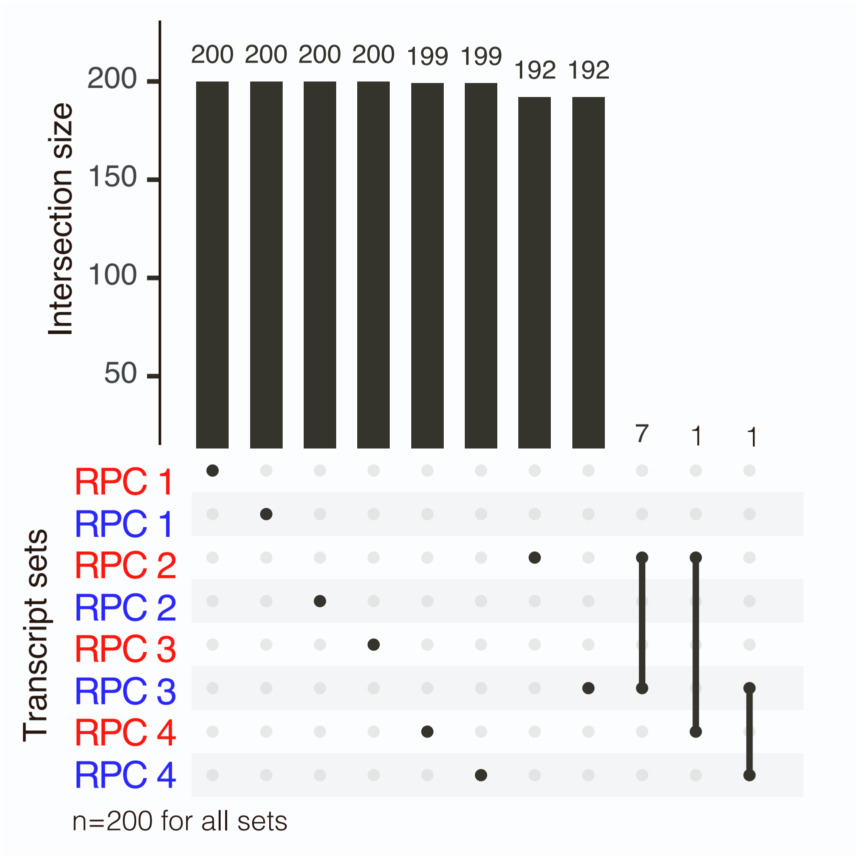

Figure S4. Upset plot showing the overlap between the top largest (200 positive and 200 negative) transcript weightings for each RPC, Related to Figure 5. Each row corresponds to a set. Each column corresponds to a possible intersection: the filled-in cells show which set is part of an intersection and the bar charts on the top show the number of elements in the intersection.

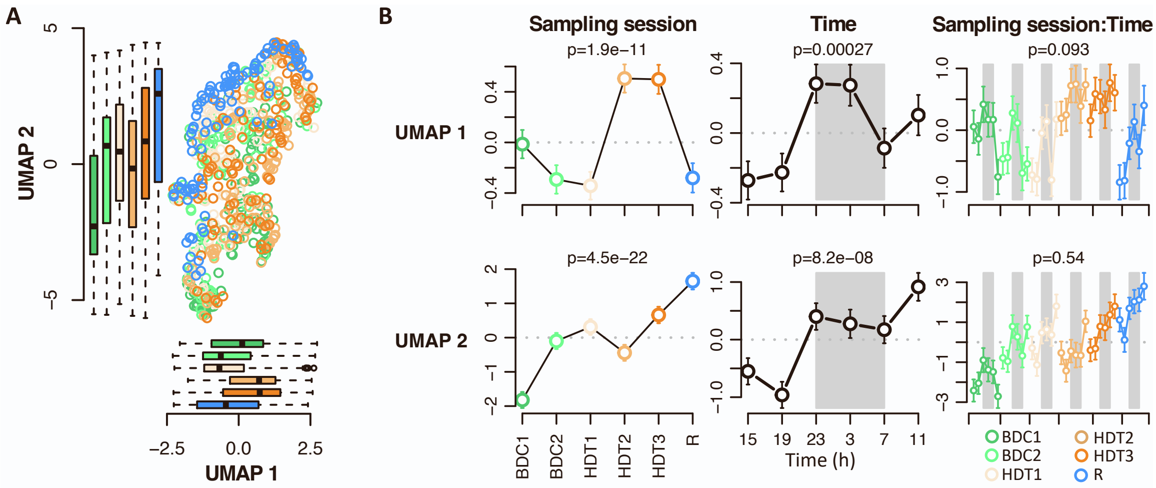

Figure S5. Dimensionality reduction via UMAP, Related to STAR Methods. A Uniform Manifold Approximation and Projection (UMAP) of all samples on layout components (UMAP1 and UMAP2). Boxplots show the distribution of samples along the two dimensions. **B** Mixed model ANOVA of layout components with main effects of sampling session (left column), time (centre column) and sampling session:time interaction (right column) for each dimension (rows, UMAP1 and UMAP2). Vertical axes correspond to the layout component least squares mean +/- std. error for sample groups shown on the horizontal axes. Groups in the right column follow a temporal order within each condition.

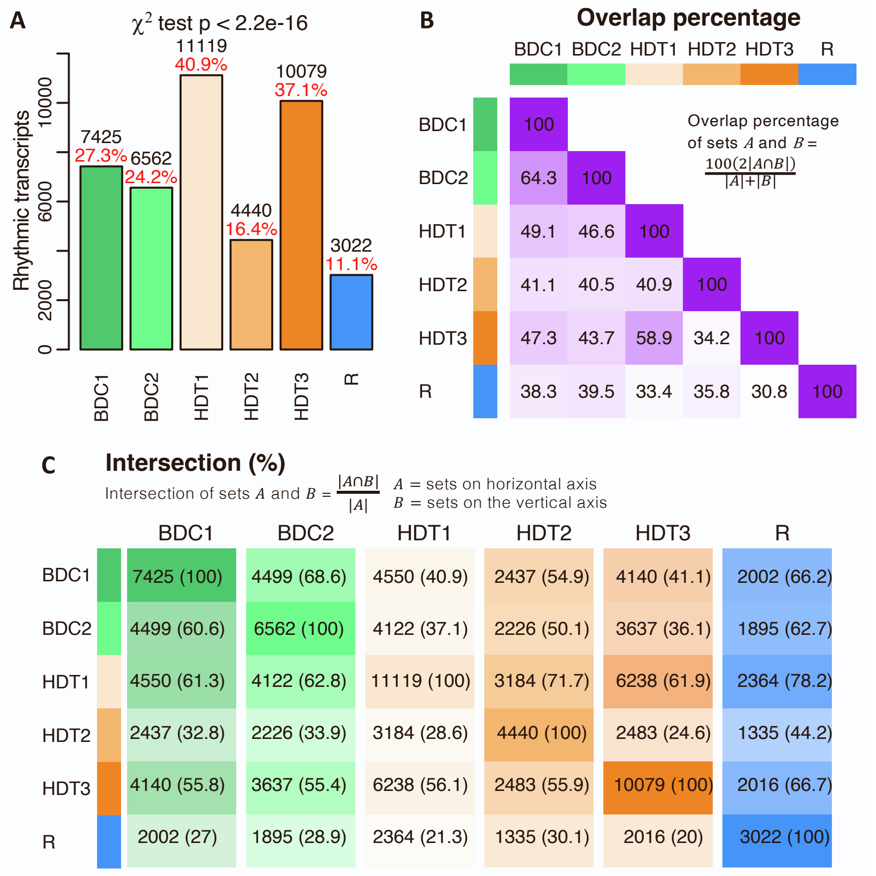

Figure S6. Rhythmic transcripts common across sampling sessions, Related to Figure 6. A Number and percentage of rhythmic transcripts for each sampling session. Percentage relative to the total number of transcripts analysed, n=27,154. The χ^2 statistic was used to compare the effect of sampling session in the number of rhythmic transcripts. **B** Percentage of overlap of rhythmic transcripts between sampling sessions. Purple colour map reflects percentage of overlap with 100% overlap as dark purple and 0% overlap as white. **C** Proportion of rhythmic transcripts in one sampling session that is overlapped by rhythmic transcripts from another sampling session. One colour map per column is used with dark colour representing 100% and white 0%. All results shown are from rhythmic modelling of transcripts based on a mixed-model with subject-specific MESORs.

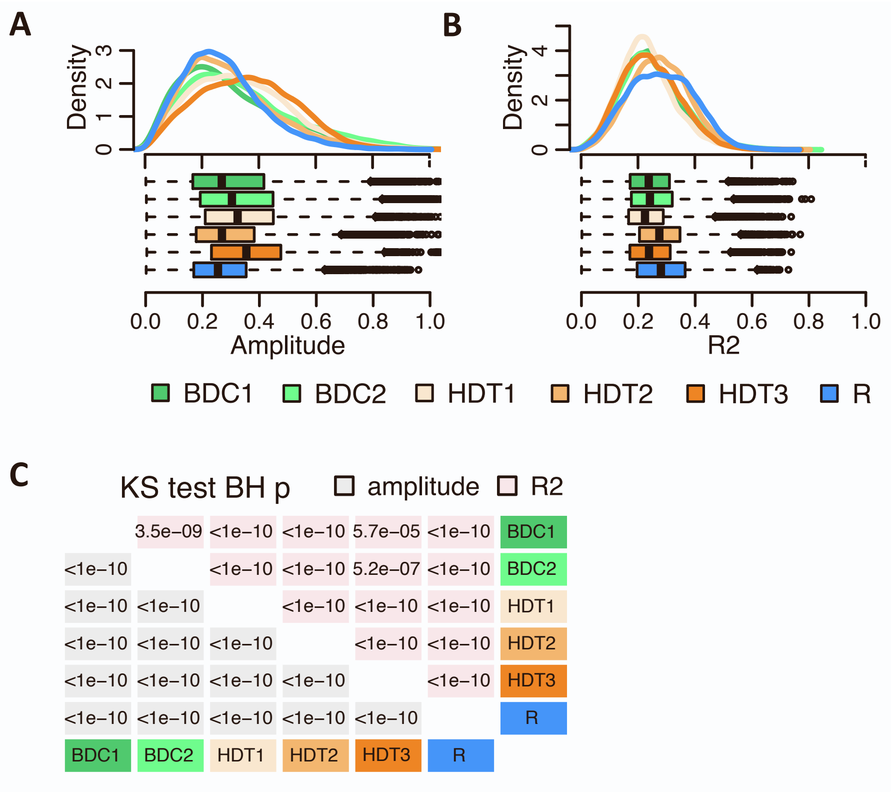

Figure S7. Amplitude and R^2 fit for all transcripts, Related to Figure 7. A Rhythmic amplitude estimate and B model R^2 distributions of all transcripts (n = 27,154). **C** All pairwise comparisons of amplitude and R^2 distributions in panels **A** and **B** using Kolmogorov-Smirnov (KS) test. Cells show Benjamini and Hochberg (BH) corrected p values for amplitude comparisons in grey and R^2 comparisons in pink. Results shown are from the rhythmic modelling of transcripts based on a mixed-model with subject-specific rhythm slopes.

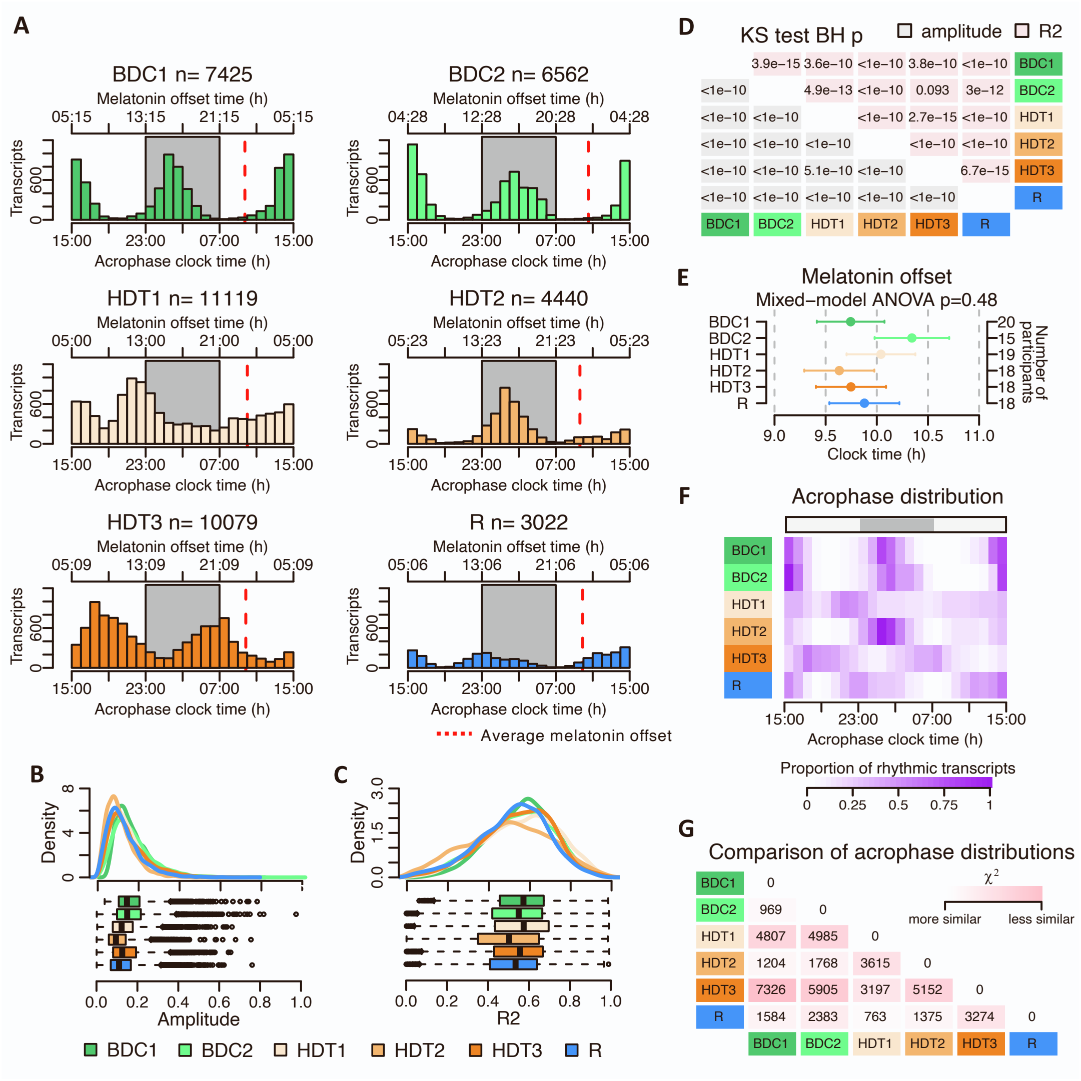

Figure S8. Characterisation of rhythmic transcripts, Related to Figure 7. A Number of rhythmic transcripts (n), acrophase distribution (bottom horizontal and left vertical axes) and average melatonin offset time (top horizontal axis, red dashed line) in the different sampling sessions. Total number of transcripts analysed is 27,154. Lights-off period is indicated by a gray rectangle. **B** Rhythmic amplitude estimate and **C** model R^2 distributions of rhythmic transcripts. **D** All pairwise comparisons of amplitude and R^2 distributions in panels **B** and **C** using the Kolmogorov-Smirnov (KS) test. Cells show Benjamini and Hochberg (BH) corrected p values for amplitude comparisons in grey and R^2 comparisons in pink. **E** Comparison of melatonin offset times (horizontal axis) across sessions (vertical axis). Plotted values are least squares mean and standard error of fitted values (mixed-model ANOVA) for the effect of session. Number of participants in each condition are displayed on the right vertical axis. **F** Heatmap of acrophase distribution of rhythmic transcripts per session. Purple colormap indicates the proportion of rhythmic transcripts per session. Lights-off period is indicated by a gray rectangle (top horizontal axis). **G** All pairwise comparisons of acrophase distribution using χ^2 test. Cells show χ^2 values. Comparisons are based on the number of transcripts in each 1-hour-acrophase-bin. All χ^2 have $p < 0.05$. All results shown are from rhythmic modelling of transcripts based on a mixed-model with subject-specific MESOR.

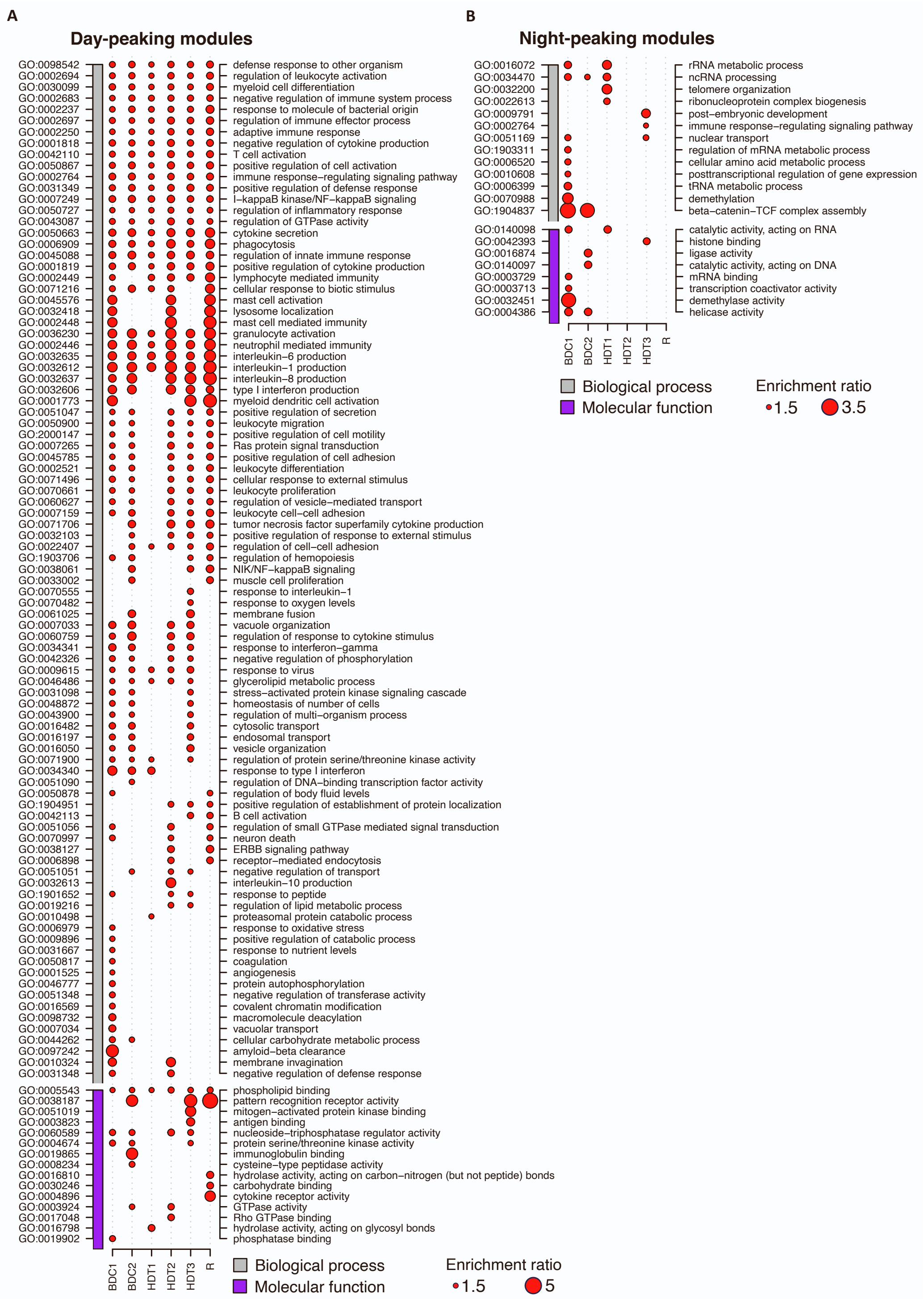

Figure S9. Functional enrichment of day and night-peaking rhythmic modules, Related to Figure 7. Gene Ontology (GO) enrichment of genes belonging to modules peaking during: **A** day (11:00 to 18:00) and **B** night (21:00 to 7:00). Vertical axes display GO terms (left) and their description (right). Biological processes and molecular functions are indicated with gray and purple vertical bars, respectively. Horizontal axis indicates sampling sessions (e.g., circle in BDC1 indicates enrichment in BDC1 modules). Circle diameter corresponds to the enrichment ratio. GO terms shown have an enrichment ratio FDR < 0.01 in at least one sampling session. Enrichment with an FDR > 0.01 are not shown. For each sampling session only top 40 terms based on p-value are considered.

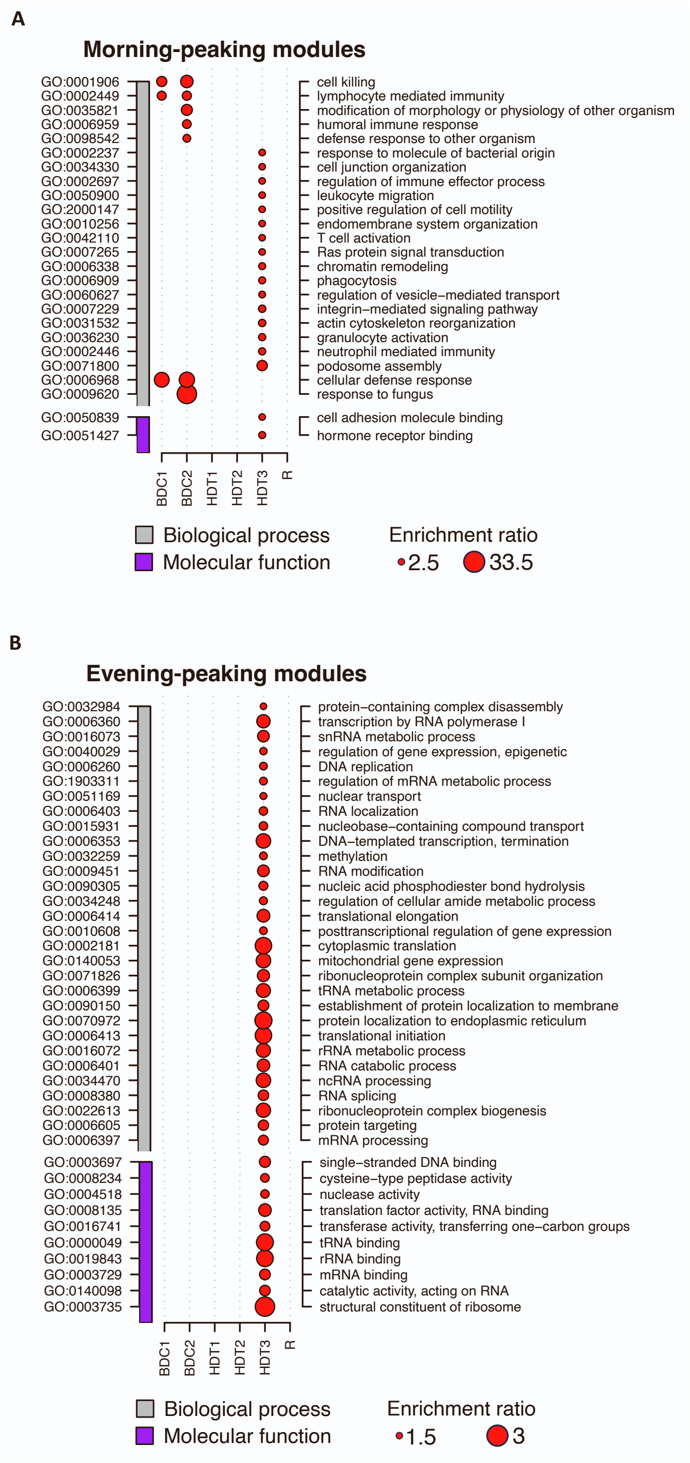

Figure S10. Functional enrichment of morning and evening-peaking rhythmic modules, Related to Figure 7. Gene Ontology (GO) enrichment of genes belonging to modules peaking during: **A** morning (07:00 to 11:00) and **B** evening (18:00 to 21:00). Vertical axes display GO terms (left) and their description (right). Biological processes and molecular functions are indicated with gray and purple vertical bars, respectively. Horizontal axis indicates sampling sessions (e.g., circle in BDC1 indicates enrichment in BDC1 modules). Circle diameter corresponds to the enrichment ratio. GO terms shown have an enrichment ratio FDR < 0.01 in at least one sampling session. Enrichment with an FDR > 0.01 are not shown. For each sampling session only top 40 terms based on p-value are considered.

Table S1. Top ten GO terms (or only those with FDR < 0.05) for the absolute value top loading transcripts for each RPC, Related to Figure 4, Figure 5.

RPC	Gene Set	Description	P value	FDR
**RPC1**	GO:0006397	mRNA processing	6.3978e-10	7.2423e-7
	GO:0008380	RNA splicing	1.0422e-8	0.000005899
	GO:0040029	Regulation of gene expression, epigenetic	0.00001019	0.0038440
	GO:0003729	mRNA binding	0.00002736	0.0077441
	GO:1903311	Regulation of mRNA metabolic process	0.00009040	0.020467
	GO:0006403	RNA localisation	0.00015065	0.028423
	GO:0071826	Ribonucleoprotein complex subunit organisation	0.00021640	0.034995
**RPC2**	GO:0036230	Granulocyte activation	<2.2e-16	<2.2e-16
	GO:0002446	Neutrophil mediated immunity	<2.2e-16	<2.2e-16
	GO:0006909	Phagocytosis	2.6645e-15	1.0054e-12
	GO:0050900	Leukocyte migration	2.1784e-10	6.1649e-8
	GO:0002764	Immune response regulating signalling pathway	7.0625e-8	0.000015989
	GO:0045730	Respiratory burst	3.3091e-7	0.000057505
	GO:0051056	Regulation of small GTPase mediated signal transduction	3.5560e-7	0.000057505
	GO:0007159	Leukocyte cell-cell adhesion	0.00000138	0.00019589
	GO:0072593	Reactive oxygen species metabolic process	0.00000345	0.00038203
	GO:0004674	Protein serine/threonine kinase activity	0.00003604	0.00038203
**RPC3**	GO:0022613	Ribonucleoprotein complex biogenesis	5.1235e-9	0.000004074
	GO:0016072	rRNA metabolic process	1.0423e-8	0.000004074
	GO:0034470	ncRNA processing	1.0798e-8	0.000004074
	GO:0140053	Mitochondrial gene expression	0.00000790	0.0022360

Table S2. Top ten GO terms (or only those with FDR < 0.05) for the top loading positive and negative transcripts for each RPC, Related to Figure 4, Figure 5.

RPC	Gene Set	Description	P value	FDR
**RPC1 negative**	GO:0006397	mRNA processing	9.8268e-10	0.000001112
	GO:0008380	RNA splicing	1.4995e-8	0.000008486
	GO:0040029	Regulation of gene expression, epigenetic	0.00000204	0.00077089
	GO:0006403	RNA localisation	0.00000516	0.0014616
	GO:0071826	Ribonucleoprotein complex subunit organisation	0.00000818	0.0018531
	GO:0003729	mRNA binding	0.00003327	0.0062778
	GO:1903311	Regulation of mRNA metabolic process	0.00010926	0.017668
	GO:0010608	Posttranscriptional regulation of gene expression	0.00028672	0.040160
	GO:0016458	Gene silencing	0.00031929	0.040160
	GO:0003725	Double-stranded RNA binding	0.00044120	0.049944
**RPC2 negative**	GO:0036230	Granulocyte activation	<2.2e-16	<2.2e-16
	GO:0002446	Neutrophil mediated immunity	<2.2e-16	<2.2e-16
	GO:0006909	Phagocytosis	2.6645e-15	1.0054e-12
	GO:0050900	Leukocyte migration	2.1784e-10	6.1649e-8
	GO:0002764	Immune response regulating signalling pathway	7.0625e-8	0.000015989
	GO:0045730	Respiratory burst	3.3091e-7	0.000057505
	GO:0051056	Regulation of small GTPase mediated signal transduction	3.5560e-7	0.000057505
	GO:0007159	Leukocyte cell-cell adhesion	0.00000138	0.00019589
	GO:0072593	Reactive oxygen species metabolic process	0.00000345	0.00038203
	GO:0004674	Protein serine/threonine kinase activity	0.00003604	0.00038203
**RPC3 positive**	GO:0022613	Ribonucleoprotein complex biogenesis	5.1235e-9	0.000004074
	GO:0016072	rRNA metabolic process	1.0423e-8	0.000004074
	GO:0034470	ncRNA processing	1.0798e-8	0.000004074
	GO:0140053	Mitochondrial gene expression	0.00000790	0.0022360
**RPC3 negative**	GO:0019787	Ubiquitin-like protein transferase activity	0.00001042	0.01180
	GO:0016684	Oxidoreductase activity acting on peroxide as acceptor	0.00004921	0.026260
	GO:0051865	Protein ubiquitination	0.00008980	0.026260
	GO:0010498	Proteasomal protein catabolic process	0.00009279	0.026260
**RPC4 positive**	GO:0048193	Golgi vesicle transport	1.0009e-7	0.00011330
	GO:0003924	GTPase activity	0.00004082	0.023107
	GO:0001882	Nucleoside binding	0.00010096	0.033368
	GO:0019001	Guanyl nucleotide binding	0.00011791	0.033368
